# Recombination Drives Genetic Diversification of *Streptococcus dysgalactiae* Subspecies *equisimilis* in a Region of Streptococcal Endemicity

**DOI:** 10.1371/journal.pone.0021346

**Published:** 2011-08-03

**Authors:** David J. McMillan, Santosh Y. Kaul, P. V. Bramhachari, Pierre R. Smeesters, Therese Vu, M. G. Karmarkar, Melkote S. Shaila, Kadaba S. Sriprakash

**Affiliations:** 1 Bacterial Pathogenesis Laboratory, Queensland Institute of Medical Research, Brisbane, Queensland, Australia; 2 Griffith Medical Research College, a joint collaboration between Griffith University and Queensland Institute of Medical Research, Brisbane, Queensland, Australia; 3 Department of Microbiology, KEM Hospital, Mumbai, India; 4 Department of Microbiology and Cell Biology, Indian Institute of Science, Bangalore, India; 5 Laboratoire de Genetique et Physiologie Bacterienne, Institut de Biologie et de Medecine Moleculaires, Faculte des Sciences, Universite Libre de Bruxelles, Brussels, Belgium; National Institute of Allergy and Infectious Diseases, National Institutes of Health, United States of America

## Abstract

Infection of the skin or throat by *Streptococcus dysgalactiae* subspecies *equisimilis* (SDSE) may result in a number of human diseases. To understand mechanisms that give rise to new genetic variants in this species, we used multi-locus sequence typing (MLST) to characterise relationships in the SDSE population from India, a country where streptococcal disease is endemic. The study revealed Indian SDSE isolates have sequence types (STs) predominantly different to those reported from other regions of the world. Emm-ST combinations in India are also largely unique. Split decomposition analysis, the presence of emm-types in unrelated clonal complexes, and analysis of phylogenetic trees based on concatenated sequences all reveal an extensive history of recombination within the population. The ratio of recombination to mutation (*r/m*) events (11∶1) and per site *r/m* ratio (41∶1) in this population is twice as high as reported for SDSE from non-endemic regions. Recombination involving the *emm*-gene is also more frequent than recombination involving housekeeping genes, consistent with diversification of M proteins offering selective advantages to the pathogen. Our data demonstrate that genetic recombination in endemic regions is more frequent than non-endemic regions, and gives rise to novel local SDSE variants, some of which may have increased fitness or pathogenic potential.

## Introduction

*Streptococcus dysgalactiae* subspecies *equisimilis* (SDSE) is a β-haemolytic Gram-positive bacterium that typically colonises the oropharynx and skin of humans. The species is closely related to *S. pyogenes*, a significant human pathogen [1]. Although generally a less common cause of human infection, SDSE causes many of the same diseases as *S. pyogenes*, including pharyngitis, pyoderma, post-glomerulonephritis, bacteraemia and other invasive diseases [2], [3]. The incidence of SDSE disease is also reported to be increasing, and in some studies exceeds that of *S. pyogenes* disease [4], [5], [6].

SDSE and *S. pyogenes* share in common many virulence factors that contribute to virulence, including the M-protein [7], [8], [9]. The M-protein protects bacteria from opsonophagocytosis by blocking deposition of complement on the bacterial surface. Nucleotide variation in *emm*, the gene encoding the M-protein, is used to type both SDSE and *S. pyogenes* at the subspecies level. Currently more than 50 SDSE *emm*-genes are present in the Centre for Disease Control *emm*-gene database (http://www.cdc.gov/ncidod/biotech/strep/types_emm103-124.htm). Other typing methods such as vir-typing [10], [11] and *emm*-pattern typing [12] also target the locus encoding the *emm*-gene. However several studies have reported the *emm*-gene, and surrounding loci to be subject to lateral gene transfer (LGT) [13], [14].

Recovery of SDSE from the throats has been reported to exceed that of *S. pyogenes* in regions where streptococcal disease is endemic [15], [16]. The diversity of circulating SDSE and *S. pyogenes emm*-types in endemic regions is high. Multilocus sequence typing (MLST) is a nucleotide based method for characterising genetic relationships amongst isolates of the same bacterial species. Unlike *emm*-typing, MLST utilises multiple housekeeping genes considered to be selectively neutral that are located in different parts of the genome. MLST therefore provides a better tool for determination evolutionary relationships within the SDSE population than typing using *emm* gene, which is under strong diversifying selective pressure. Recent MLST studies of SDSE isolates from Australia, Portugal and USA reported a high degree of genetic diversity in these populations, and revealed LGT of housekeeping alleles was occurring [14], [17]. In the current study we have used MLST to assess the genetic diversity of SDSE recovered from India, a country where streptococcal disease is endemic [18], [19] and *emm*-type diversity is high [15], [20]. Our results demonstrate this geographically confined collection to contain predominantly novel sequence types (STs). The ratio of recombination to mutation in house-keeping alleles in this endemic region surpasses that reported for non-endemic regions [17]. The data additionally suggests that LGT between SDSE and other streptococcal species occur. Our findings suggest an evolutionary process in which novel genetic variants of SDSE, possibly with altered fitness or pathogenic potential, are more likely to arise in endemic regions than non-endemic regions.

## Results

### Allelic variation

Details of the ST and *emm*-type of the 181 SDSE isolates from India are provided in Table S1. A summary of nucleotide variation in the seven loci used for MLST in SDSE is provided in Table 1. The total number of alleles present at each locus varied from six for *gtr* to sixteen for *xpt*. Ten of the new SDSE MLST alleles identified in the study were identical to alleles from GAS (Table S2). Another allele, *recP22*, was identical to a nucleotide sequence from *S. agalactiae*, and shares greater than 99% identity with the same sequence in two other *S. agalactiae* genomes. As *recP22* is less than 90% identical to other so far known SDSE and *S. pyogenes recP* alleles, the allele was most likely acquired from *S. agalactiae*. Although evidence for mobile genetic element (MGE) mediated LGT between SDSE and *S. agalactiae* has been reported [21], to our knowledge this is the first evidence suggesting lateral transfer of an *S. agalactiae* housekeeping gene to SDSE.

**Table 1:** Sequence variation in SDSE MLST loci from India.

Gene	Size of partial gene	Total alleles	New alleles	New non-SDSE alleles	nt variant positions	π^a^	d_n_	d_s_	d_n_/d_s_
*gki*	498	8	3	3	14	0.011	0.011	0.043	0.247
*gtr*	450	6	2	2	4	0.004	0.004	0.006	0.678
*murI*	438	9	4	2	15	0.015	0.008	0.036	0.222
*mutS*	405	9	6	1	6	0.006	0.002	0.019	0.081
*recP*	459	14	4	2	31	0.034	0.007	0.150	0.041
*xpt*	450	16	8	0	28	0.020	0.005	0.071	0.067
*atoB*	434	10	5	1	47	0.042	0.033	0.067	0.486

a Nucleotide diversity, d_n_ and d_s_ values were determined using alleles unique to SDSE. i.e. alleles likely acquired from *S. pyogenes* and *S. agalactiae* were excluded.

When MLST alleles predicted to have been acquired by SDSE from non-SDSE sources through recombination (defined below) were disregarded, nucleotide diversity ranged from 0.004 for *gtr* to 0.042 for *atoB*. With the exception of *gtr* and *atoB*, the d_n_/d_s_ ratio was less than 0.4 for all loci, indicating that the variation observed in these alleles is likely constrained by purifying selection. Although the d_n_/d_s_ for *gtr* was relatively high compared to the other MLST alleles, nucleotide variation only occurred at four sites. The number of variable sites for the other MLST alleles ranged from 6 to 47. Phylogenetic analysis of the SDSE *atoB* and its orthologue in GAS (*yqiL*), showed two SDSE alleles, *atoB16* and *atoB17*, which accounted for the majority of non-synonomous mutations, lie at a node midway between the SDSE and GAS clusters (Figure S1).

### Clonal relationships in the Indian SDSE population

**Figure 1:**
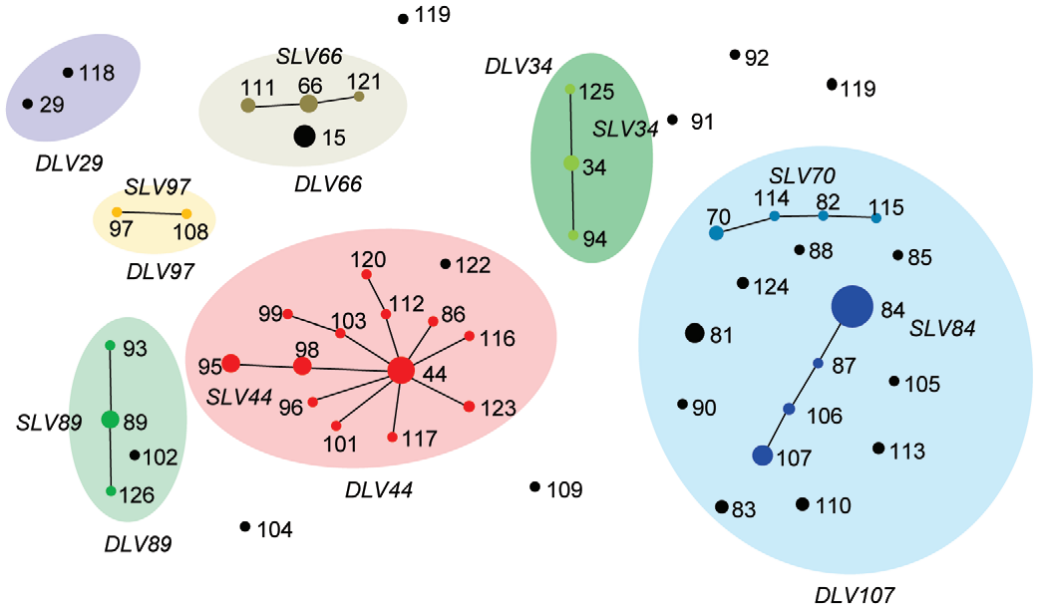
Population structure of Indian SDSE isolates. The 52 STs clustered into seven SLV-defined CCs. STs part of the same SLV defined CC are joined by black lines. When grouped at the less stringent DLV level seven CCs are defined (light shaded circles).

MLST resolved the 181 isolates into 52 STs. The most common ST, ST84, was found in 43 isolates, all of which were *emm*-type stg4831. The five next most abundant STs (ST44, ST15, ST89, ST81 and ST107) each individually constitute 5 to 10% of the total collection, and together with ST84 account for 53% of isolates. In contrast, thirty two STs (62%) are represented by a single isolate. The overall diversity of *emm*-type (*D* = 0.898, 95% CI: 0.872–0.924) and ST (*D* = 0.916, 95% CI: 0.890–0.942) in the population is similar. The eBURST analysis segregated the 52 STs into seven single locus variant clonal complexes (CC_slv_s), and twenty singletons (Figure 1). CC_slv_44 contained the greatest number of STs (n = 13). CC_slv_84 contained the greatest number of isolates (n = 43), and together with CC_slv_44 (n = 41), account for 58% of all isolates. No other CC_slv_ contained more than 15 isolates. When clustered at the DLV (CC_dlv_) level, seven complexes, and six singletons were defined. Together CC_dlv_44 and CC_dlv_107 account for 70% of isolates. Overall, while a large proportion of SLV defined singletons became a part of CC_dlv_s, the CCs_lv_s themselves remained largely discrete.

**Figure 2:**
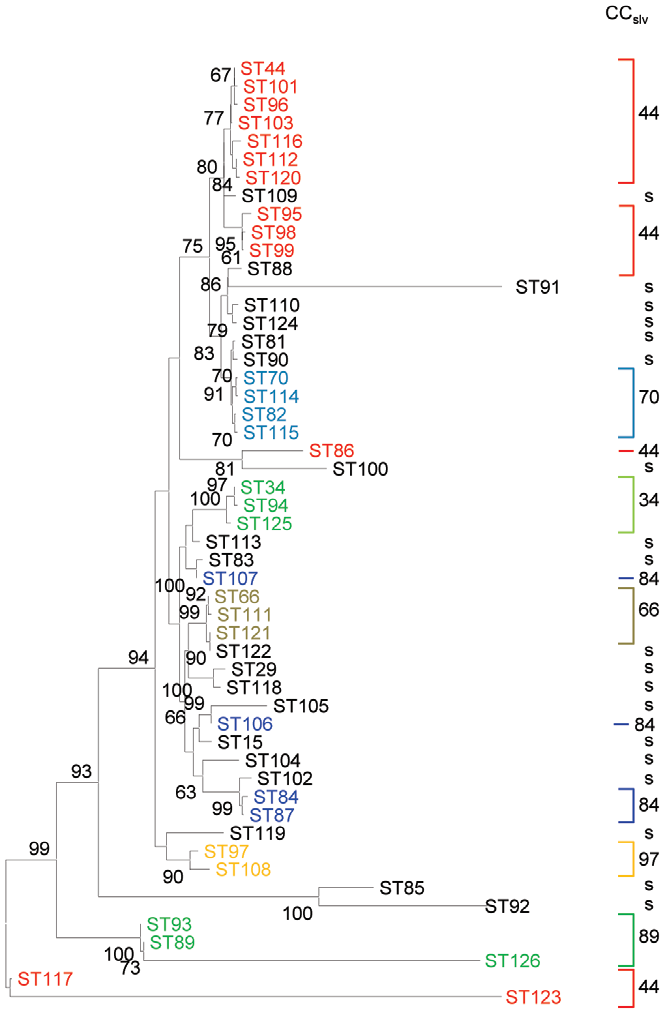
Minimum evolutionary tree of concatenated SDSE MLST loci. The tree was constructed using concatenated sequences of 52 SDSE STs. The tree is drawn to scale, with branch lengths in the same units as those of the evolutionary distances used to infer the phylogenetic tree. Only bootstrap values greater than 80% are shown. Clonal complexes at both the SLV and DLV level, and singletons (s), as determined using eBURST, are also depicted.

Relationships between STs were examined at the nucleotide level by constructing Minimum Evolution (ME) spanning trees using concatenated sequences of all seven MLST loci. In general, there is a good congruence between relationships of STs within CC_slv_s, and the clustering of STs within individual branches of the ME tree (Figure 2). The branches of the ME tree were subsequently partitioned at nodes separating STs belonging to different CC_slvs_ The grouping of STs in these partitions were then compared to the grouping of STs by eBURST at the CC_slv_ level using the Wallace co-efficient [17], [22]. The correlation between eBURST derived partition and ME derived partitions was calculated to be 0.630 (CI95% 0.388–0.872). There were five instances in the tree where STs did not cluster with the CC_slv_ as predicted by eBURST. As examples, ST117 and ST123, both of which belong to CC_slv_44, possess gki15 and gki16. These alleles are identical to the GAS MLST alleles gki67 and gki38, respectively. ST86, another ST from CC_slv_44 possesses the divergent *ato17* (Figure S1). Genetic relationships consistent with recombination were also apparent in STs that did not cluster as predicted from eBURST analysis.

### Recombination and mutation in MLST loci

Estimates of recombination and mutation in SLV pairs were determined using previously described methods [22], [23], [24]. Of the 25 SLV pairs 22 are predicted to have arisen through recombination (Table S3); three recombination events involved non-SDSE derived alleles. Only two SLV pairs are predicted to have arisen through point mutation. The *r/m* ratio in the population was 11, and per site *r/m* ratio 164. When alleles predicted to be derived from non-SDSE species were excluded the per site *r/m* ratio fell to 41. The standardised Index of Association (I_A_) across the population was 0.28, also suggestive of a high rate of recombination.

**Figure 3:**
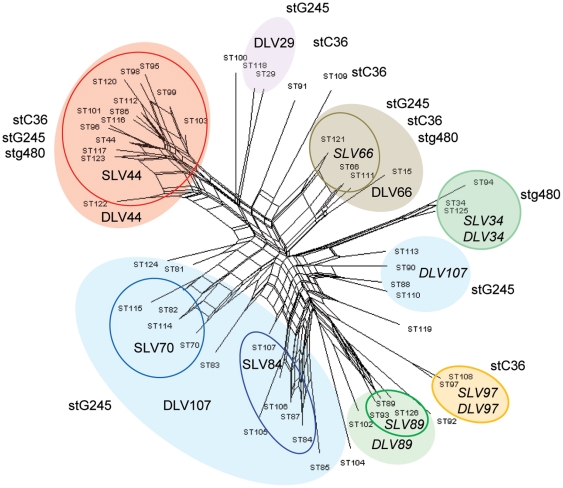
Split decomposition analysis of Indian STs. Shaded circles indicate DLV defined CCs. Solid lines enclose SLV defined CCs. STs associated with *stC36*, *stg245* and *stg480* are also indicated.

Recombination events can distort or conceal true evolutionary relationships that exist between STs. In these instances, standard phylogenetic trees, which only display single relationships between isolates or clones, do not provide the equal representation for all possible evolutionary relationships. Split decomposition analysis was therefore used to visualise alternative phylogenetic relationships between STs (Figure 3). The reticulated phylogenetic structure of this figure is indicative of extensive recombination of loci [25] providing additional support for the high estimates of recombination and low I_A_. The majority of STs were found in the same groupings as determined by eBURST. However STs belonging to DLV107 segregated into two separate groups.

### Relationship between *emm*-type and ST

*emm*-typing is the most common method of typing SDSE. Discrepancies between *emm*-type and ST have previously been reported [17] and offers evidence that LGT of *emm*-genes occurs in nature [26]. The presence of multiple *emm*-types within a single ST that are identical to, or have close similarity to emm-genes found in distant STs is suggestive of LGT of this gene [17], [27]. Alternatively, diversifying selection pressure on the emm-gene may also give rise to STs that harbour multiple emm-types. In this study, eleven STs were associated with more than one *emm*-type (Table 2). Two of these, ST44 and ST15, were each associated with five *emm*-types. Together nineteen instances of multiple emm-genes present in individual STs was observed. A complementary method for inference of LGT is the identification of the same *emm*-gene in distantly related or unrelated STs. In total eleven *emm*-genes were found in two or more CC_dlv_s (Table S4). Four *emm* genes, *stc36*, *stg485*, *stg480* and *stg866* were present in 4, 4, 3 and 3 CC_dlv_s respectively (Figure 3). Seventeen instances of *emm*-gene LGT were inferred using this method. In contrast to the frequency of recombination of the *emm*-gene, the predicted number of recombination events for individual housekeeping alleles used in MLST ranged from one for *xpt* to six for *murI* based on SLV relationships.

**Table 2:** Sequence types associated with multiple *emm*-types.

ST	Emm-type
15	*stc36, stg10, stg245, stg866, stgL265*
29	*stc74a, stg245*
34	*stg480, stgm22*
44	*stc36, stg245, stg480, stg6, stgL265*
66	*stC1400, stg6792*
81	*stg245, stg6*
83	*stc74a, stg245*
95	*stg653, stg6972*
98	*stc36, stg2078, stg480*
107	*emm23, stC5345, stc6979*
110	*stg1750, stg245*

**Figure 4:**
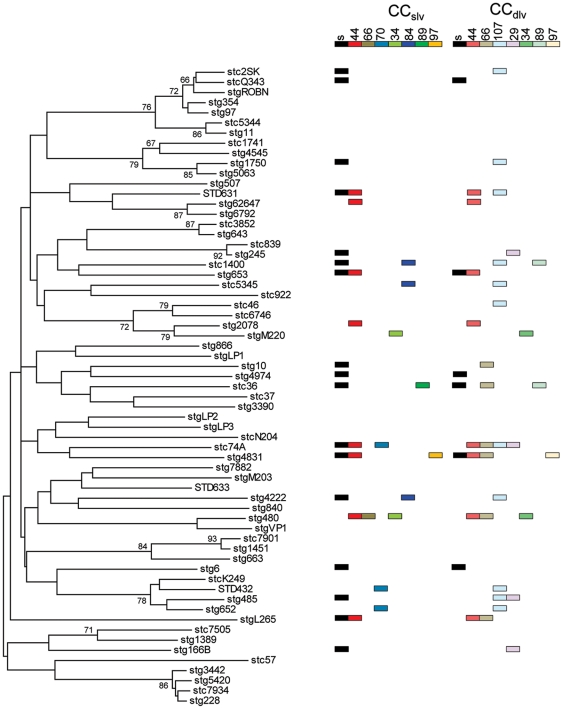
Phylogenetic relationship of SDSE based on *emm* nucleotide sequence. The tree was constructed using the ME algorithm. Bootstrap values in which associated taxa are clustered in greater than 60% of cases are shown next to branches. CC_slv_s and CC_dlvs_ associated with emm-types are also shown.

To further investigate the relationships between *emm*-type and ST, we constructed a phylogenetic tree using the 150 nucleotides of the emm-gene used to determine emm-type (Figure 4). The *emm*-gene sequences were aligned using ClustalW prior to construction of the ME tree. When MLST data was overlaid onto this tree, CC_slv_s and CC_dlv_s were scattered throughout the tree. When *emm*-types were mapped onto the SplitsTree phylogram (which accommodates effects of recombination), a much clearer association between *emm*-type and MLST data became apparent, with the majority of isolates of the *emm*-types falling into the same cluster (Figure 3). These clusters are therefore the likely progenitors of specific emm-genes.

### Indian isolates possess unique STs

Eighty STs were described in a previous study of 178 SDSE isolates from non-endemic regions [17]. Only six of these STs (ST15 ST29 ST34 ST44 ST66 ST70) were found amongst the Indian strains. Of the 46 new STs in this study, nineteen possessed previously reported alleles at all seven loci, but in combinations not previously found. Twenty-seven STs possessed at least one new allelelic sequence. When all known SDSE STs were compared using eBURST, seven CC_dlv_s were defined (Figure S2). One of these complexes was considerably larger than all others with 87 STs. With the exception of four STs (ST97, ST108, ST100 and ST104), all Indian STs were found in this large complex. In contrast only 43 of the 74 non-Indian STs were found within this complex. Further comparisons using split decomposition (Figure S3) and ME analysis (Figure S4) also segregated Indian isolates into subclusters separate from other SDSE isolates.

### Association between disease and ST

Chi-squared goodness-of-fit tests were used to determine if any ST or CC was over-represented in strains recovered from individuals with throat infection,(i.e. presenting with pharyngitis or tonsillitis) when compared to strains recovered from individuals without symptoms of throat infection [15]. The latter control group included all SDSE throat carriage isolates, and SDSE recovered from the skin. Three STs were found to have statistically different distributions between throat-infection and control isolates. ST89 and ST111 were found to be overrepresented in the throat infection isolates (relative risk of 2.61 and 2.85 respectively). The third ST, ST107 was underrepresented (relative risk of 1.63 for non-throat infection). No CC had an increased association with throat infection.

## Discussion

Streptococcal infection is considered to be endemic in India. As a consequence co-infection with multiple SDSE strains, or SDSE and other streptococcal species, is more likely to occur here than in countries where streptococcal disease is non-endemic. An outcome of increased inter-strain contact is increased opportunity for LGT, resulting in an increase in overall strain diversity. The ratio of recombination to mutation reported here is double that reported for SDSE from non-endemic areas, supporting this scenario [17]. Further the inter-relatedness of Indian isolates and presence of only a few global STs, support a model whereby ongoing recombination between isolates in a single geographic location result in strains that over time become more related to each other than to strains from outside of the population.

In our study, recombination involving the *emm*-gene was more frequent than recombination involving individual housekeeping alleles. The *emm*-gene is part of an ancient pathogenicitiy island that is mobilisable *in vitro* [13]. An active mechanism for LGT involving the emm-gene therefore exists, and may account for the increased LGT of this gene. The acquisition of new emm-genes that assist SDSE in evasion of host immune responses, giving recipients a selective advantage, may also increase the frequency in which LGT involving the emm-gene is observed when compared to selectively neutral house-keeping genes. In spite of this, we observed more *S. pyogenes* housekeeping alleles than *S. pyogenes emm*-genes among our SDSE isolates. This suggests restricted compatibility for the *S. pyogenes emm* genes in SDSE, and may explain distinct evolutionary clades for the genes in the two species. The amino-termini of the mature M-proteins are the major target of type-specific anti-GAS and anti-SDSE antibodies. While population and virulence studies of *S. pyogenes* report associations between *emm*-types and specific disease, associations between *emm*-type and SDSE-mediated disease are not obvious [28], [29], [30]. One outcome from our study that could account for this observation is that the greater recombination occurring among SDSE isolates in regions where streptococcal infection is endemic results in the weakening of emm-typing as a useful tool for assigning genetic relationships over a meaningful time frame amongst geographically separated SDSE isolates. Of the six STs shared between India and the rest of the world, only five *emm*-STs were common. Twelve *emm*-STs were only found in India, and another nine *emm*-STs found in non-Indian isolates.

One *emm*-type, *stg480*, has been associated with SDSE infection in several studies [5], [31]. This *emm*-type was also one of the most commonly recovered *emm*-types in this study, but was not recovered more frequently from individuals with pharyngitis. The apparent increased association with diseases reported in other studies may reflect the relative abundance of this *emm*-type within the population, rather than an increased virulence potential. *stg480* has now been associated with five STs in this and previous studies [14], [17], suggesting that this *emm*-locus has propensity to participate in LGT frequently, which indeed further clouds epidemiological findings based on *emm*-type. Larger prospective studies that include characterisation of both the *emm*-gene and ST are required to determine the pathogenic potential of individual SDSE lineages.

In contrast to *stg480*, the majority of *stg4831* isolates were predominantly associated with ST84. All ST84 isolates also possessed the *stg4831 emm*-gene. As all ST84 and *stG4831* isolates were only recovered in Mumbai, it is likely that during the collection period, an outbreak of *stg4831*-ST84 was occurring. The general lack of variation in ST-*emm* combinations suggests this outbreak is relatively new, leaving little time for mutation or recombination with existing strains. Nevertheless, the presence of three stG4831 clones with different ST (ST87, ST85 and ST102), all recovered from Mumbai, once again suggests that recombination was occurring at a local level. ST87 is an SLV of ST84, predicted to have arisen *via* recombination. ST85, a DLV variant of ST84 is more closely related to ST84 than any other ST. Two of the three alleles that differ between ST102 and ST84 are found in ST89. In this instance, the data suggest recombination between an ST84 clone and isolate from CC_slv_89 has occurred.

Frequent recombination is emerging as a paradigm for the β-hemolytic streptococci. Taken together our findings suggest that SDSE ST diversity is high in regions where streptococcal disease is considered endemic, and is driven mainly by recombination. In endemic regions, the opportunity for different streptococcal isolates to come into contact, and share genes is greater than in regions where streptococcal infection is less prevalent. SDSE, *S. pyogenes* and *S. agalactiae* all possess multiple MGEs, and MGE-related interspecies transfer of virulence genes has been reported by several groups [26], [32], [33], The transfer of non-MGE housekeeping genes may occur as bystander event during transfer of MGEs [34]. It is noteworthy that transfer of allelic variants of virulence genes not associated with mobile genetic elements is also occurring, possibly having a greater impact on the fitness or virulence of some SDSE isolates. The observed increase in the rate of recovery of new variants in endemic regions suggests rapid emergence of more fit or virulent clones are more likely to occur in these regions.

## Materials and Methods

### Ethics statement

Ethical approval for swabbing of individuals in the study was granted by the Seth G. S. Medical School and KEM Hospital Ethic Committee, India (EC/Gov/-4/2006). Written informed consent for the swabbing of children was obtained from the guardians of all children included in the study.

### Bacterial Strains

A total of 181 SDSE isolates collected as part of community and school surveys carried out in Mumbai [15] and Chennai [35] were used in the study. Eighty-five isolates were collected from individuals presenting with pharyngitis (n = 66) or tonsillitis (n = 10), and were classified as ‘throat-infection’ isolates. Another 102 isolates were recovered from the throats of individuals lacking clinical signs of streptococcal infection. Nine SDSE isolates were collected from the skin of individuals presenting with pyoderma. All isolates were classified as SDSE on the basis of β-hemolytic activity, expression of the group C or G carbohydrate, and possession of characteristic *emm*-type and molecular markers characteristic of SDSE [10]. *Emm*-types for these isolates were previously reported [15], [35] or determined using standard protocols [20], [36]. Full details of strains used in this study are provided in Table S1.

### MLST

The SDSE MLST scheme based on the following seven gene targets (glucose kinase (*gki*), glutamine transport protein (*gtr*), glutamate racemase (*murI*), DNA mismatch repair protein (*mutS*), transketolase (*recP*), xanthine phosphoribosyl transferase (*xpt*) and acetoacetyl-coathioloase (*atoB*)) has been described previously [17]. With the exception of *atoB* these alleles are the same as used in the GAS MLST scheme [27]. DNA was extracted using the QIAGEN DNAeasy kit (QIAGEN, Australia), and 450–500 base-pair internal fragments of these genes were amplified under the following conditions; 2 min denaturation at 95°C, followed by 35 cycles of 95°C (45 s), 50°C (45 s) and 72°C (60 s). PCR products were purified using ExoSAPIT (USB Corp, USA), and sequenced in the forward and reverse directions by Macrogen (Korea) or in-house. Sequencher (Genecodes, USA) was used for initial analysis and trimming of sequences to match reference sequences. All sequences were compared to existing SDSE MLST alleles to determine specific allele number at a given locus. Unique allelic sequences identified in this study were assigned a new allele number. The combination of seven allele numbers was then used to determine the sequence type (ST). goeBURST (http://goeburst.phyloviz.net/) was used to identify related STs [37], [38]. In this study Clonal Complex (CCs) were defined as a group of STs that are related to each other at the Single Locus Variant (CC_slv_) or Double Locus Variant (CC_dlv_) level.

### Recombination and mutation

Rates of recombination and mutation that give rise to SLV pairs were estimated as previously described [22], [23], [24]. Briefly, SLV pairs that contain greater than one nucleotide difference in the variant alleles were classified as arising through recombination. SLV pairs in which one ST contains a unique single nucleotide polymorphism not found in other STs were classified as a point mutation event. Single nucleotide changes in SLVs pairs giving rise to alleles already present in unrelated STs in different CC_slv_s were classified as recombination events. Both the ratio of recombination/mutation events (*r/m*) and per nucleotide site ratio of recombination/mutation are reported.

### Phylogenetic Analysis

Nt diversity (π), nonsynonymous (d_n_) and synonymous substitution rates (d_s_) were calculated using DnaSP (version 5) [39]. Linkage equilibrium, expressed as standardised Index of Association (I_A_) was calculated using LIAN 3.5 [40]. Distance matrices for phylogenetic analysis were calculated using START [41]. Phylogenetic networks were constructed using Splitstree 4 [25]. Minimum evolution (ME) spanning trees using concatenated nucleotide sequences from all seven MLST loci were constructed in Mega4 [42], with support for branches provided by bootstrapping (n = 1000). Phylogenetic analysis of the 180 nt variable region of the *emm*-gene used for *emm*-typing was also performed using Mega4. The sequences were aligned using ClustalW, using default parameters, prior to construction of the ME tree. The Wallace co-efficient [23], [24] was calculated using www.comparingpartitions.info.

### Statistical analysis

Statistically significant associations between ST, Clonal Complex and other epidemiological factors were assessed using the Chi-squared goodness of fit test (*p*<0.05). Confidence intervals for Simpson index of diversity (D) were calculated as previously described [43].

## Supporting Information

Figure S1 **Phylogenetic analysis of SDSE** ***atoB*** **and S.** ***pyogenes yqiL*** **alleles.** The relationship between alleles was inferred using the Minimum Evolution method, and support for branches provided by bootstrapping (n = 1000). Bootstrap values are only provided for branches with greater than 50% support.
(TIF)

Figure S2 **eBURST analysis of all known SDSE STs.** Blue circles represent STs only found in India. Red circles represent STs found outside of India (McMillan et al., 2010). Green circles represent STs found in both collections. Dark connecting lines join SLV related pairs. Grey lines connect DLV related pairs.
(TIF)

Figure S3 **Split decomposition of all SDSE STs.** STs found in India are circled.
(TIF)

Figure S4 **ME tree of all SDSE STs.** The tree is drawn to scale, with branch lengths in the same units as those of the evolutionary distances used to infer the phylogenetic tree. Only bootstap values greater than 50% are shown. Blue circles represent STs only found in India. Red circles represent STs found outside of India (McMillan et al., 2010). Green circles represent STs found in both collections.
(TIF)

Table S1 **Details of SDSE isolates used in this study.**
(DOC)

Table S2 **Novel MLST alleles in the Indian SDSE population.**
(DOC)

Table S3 **Recombination and mutation in SDSE.**
(DOC)

Table S4 ***Emm*** **-types associated with multiple Sequence Types.**
(DOC)
