# Correction: Recombination Drives Genetic Diversification of *Streptococcus dysgalactiae* Subspecies *equisimilis* in a Region of Streptococcal Endemicity

**DOI:** 10.1371/annotation/fbdb6697-5b22-4ac5-b38a-b55fc6f86beb

**Published:** 2012-04-26

**Authors:** David J. McMillan, Santosh Y. Kaul, P. V. Bramhachari, Pierre R. Smeesters, Therese Vu, M. G. Karmarkar, Melkote S. Shaila, Kadaba S. Sriprakash

This study used genomic DNA that was derived from a collection of bacterial isolates sourced from Chennai. The authors wish to acknowledge and thank Dr Thangam Menon of the Department of Microbiology, University of Madras, Chennai, India for providing these isolates. In addition, the citation for reference 35 as one of the sources of the isolates is incorrect; none of the isolates supplied by Dr Menon for this study had previously been described in reference 35. 

