# Supplementary figures and images for: Recombination Drives Genetic Diversification of Streptococcus dysgalactiae Subspecies equisimilis in a Region of Streptococcal Endemicity
Source: PLoS One. 2011 Aug 3;6(8):e21346. doi: 10.1371/journal.pone.0021346 (PMC3153926; doi:10.1371/journal.pone.0021346)

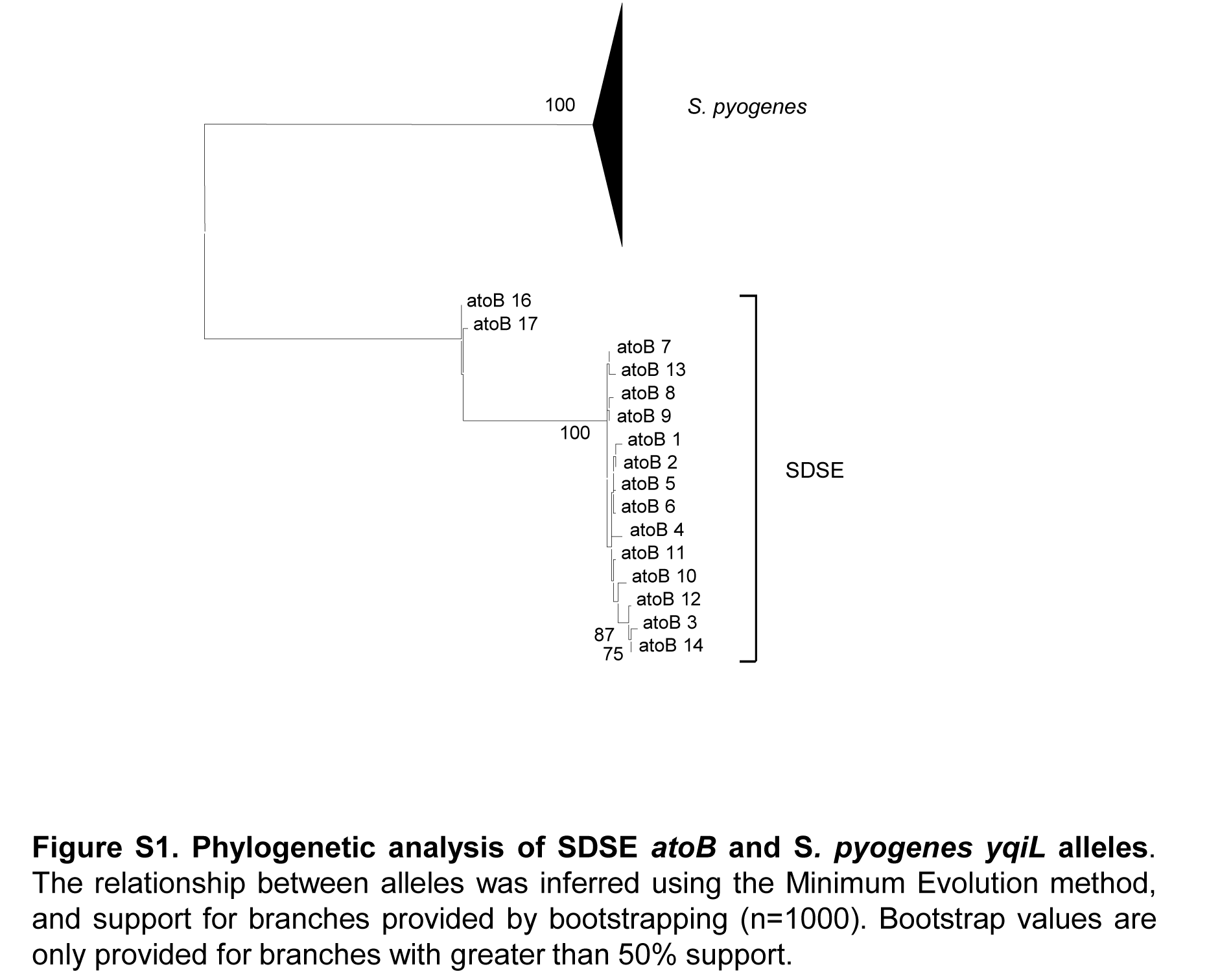

Supplement: Figure S1 — Phylogenetic analysis of SDSE atoB and S. pyogenes yqiL alleles. The relationship between alleles was inferred using the Minimum Evolution method, and support for branches provided by bootstrapping (n = 1000). Bootstrap values are only provided for branches with greater than 50% support. (TIF) [file pone.0021346.s001.tif]

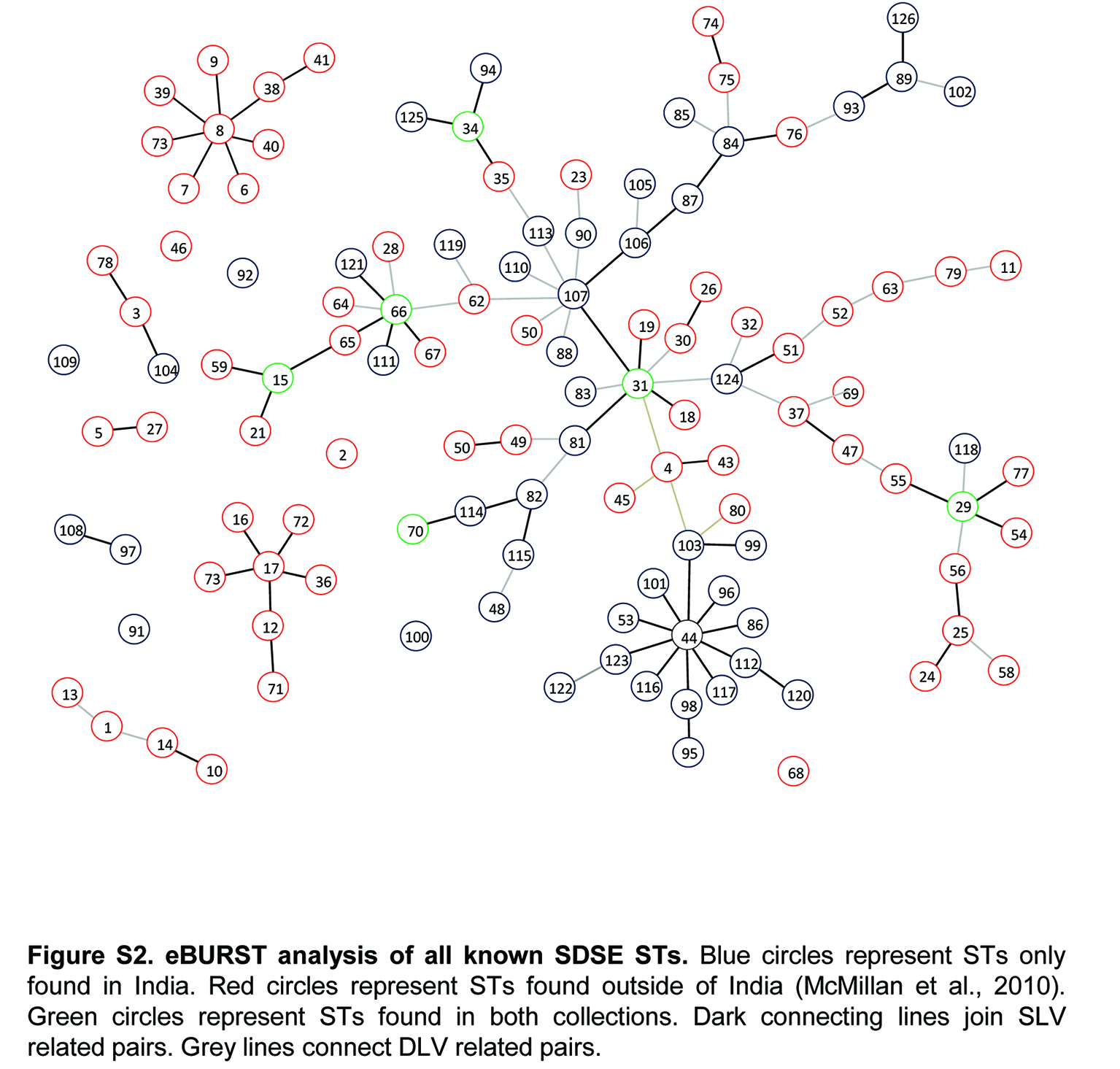

Supplement: Figure S2 — eBURST analysis of all known SDSE STs. Blue circles represent STs only found in India. Red circles represent STs found outside of India (McMillan et al., 2010). Green circles represent STs found in both collections. Dark connecting lines join SLV related pairs. Grey lines connect DLV related pairs. (TIF) [file pone.0021346.s002.tif]

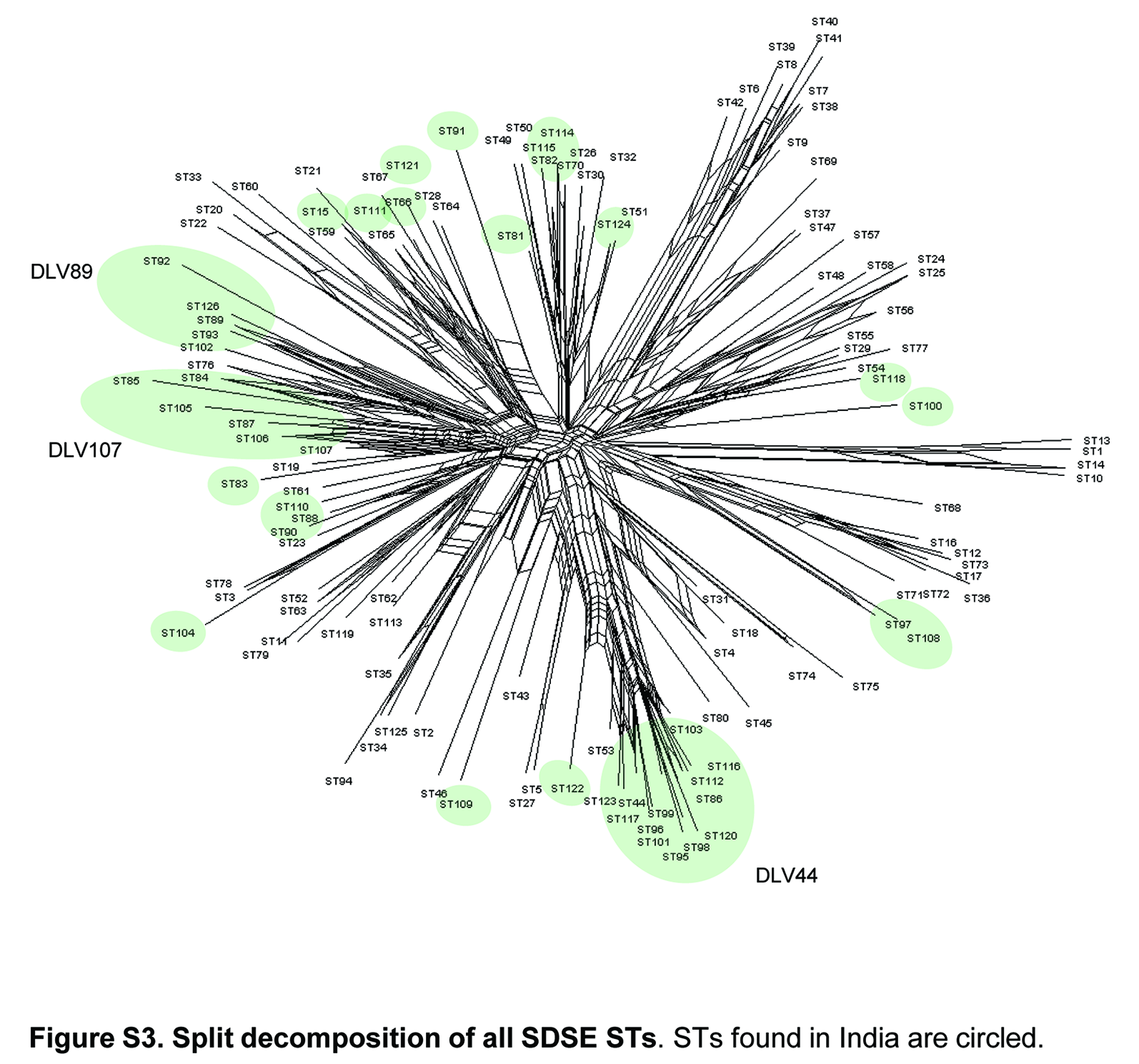

Supplement: Figure S3 — Split decomposition of all SDSE STs. STs found in India are circled. (TIF) [file pone.0021346.s003.tif]

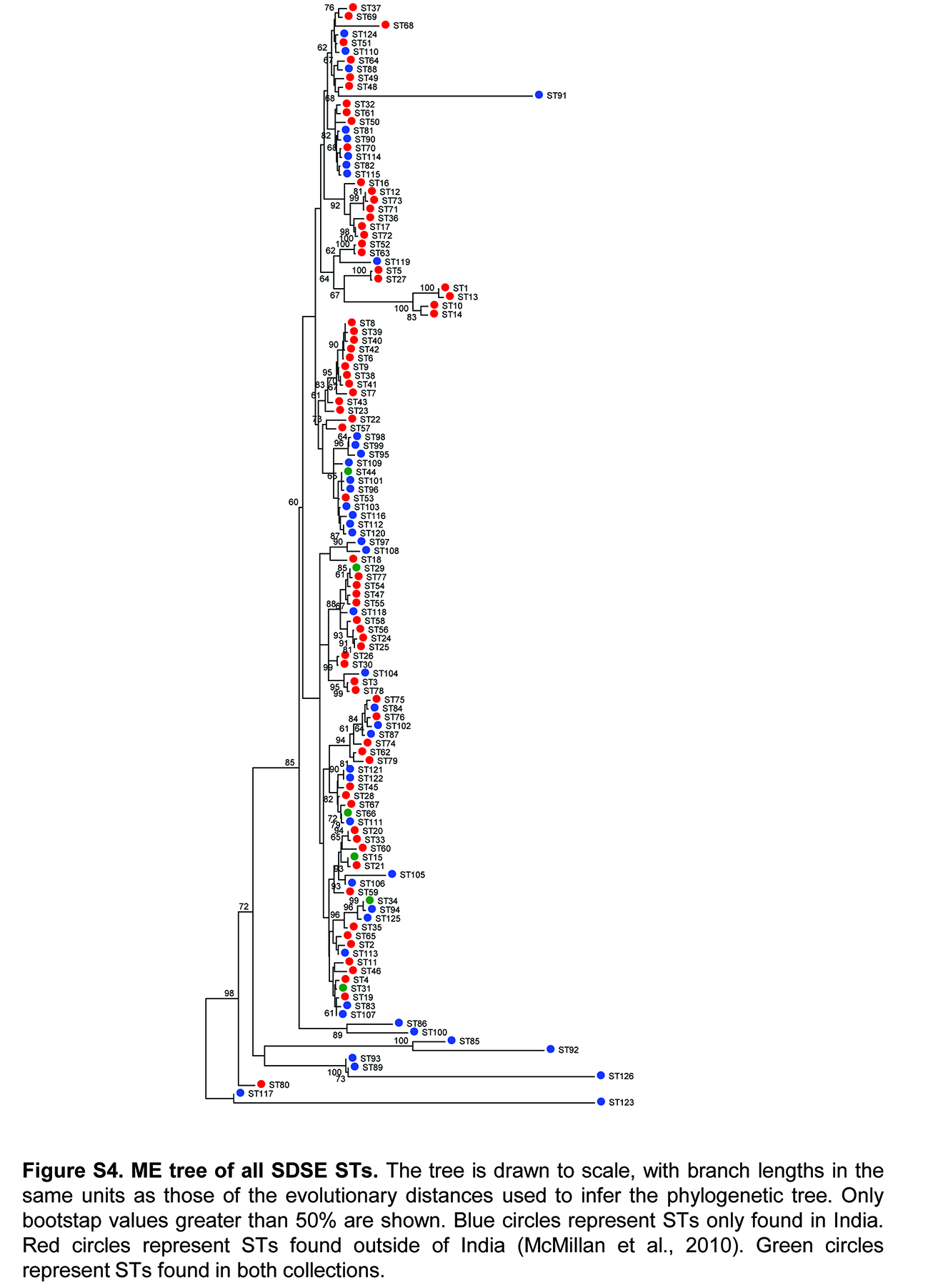

Supplement: Figure S4 — ME tree of all SDSE STs. The tree is drawn to scale, with branch lengths in the same units as those of the evolutionary distances used to infer the phylogenetic tree. Only bootstap values greater than 50% are shown. Blue circles represent STs only found in India. Red circles represent STs found outside of India (McMillan et al., 2010). Green circles represent STs found in both collections. (TIF) [file pone.0021346.s004.tif]
