# Supplementary material for: Recombination Drives Genetic Diversification of Streptococcus dysgalactiae Subspecies equisimilis in a Region of Streptococcal Endemicity
Source: PLoS One. 2011 Aug 3;6(8):e21346. doi: 10.1371/journal.pone.0021346 (PMC3153926; doi:10.1371/journal.pone.0021346)
Supplement: Table S1 — Details of SDSE isolates used in this study. (DOC) [file pone.0021346.s005.doc]

**Table S1**. Details of SDSE isolates used in this study

| **Strain** | **Emm-type** | **Source** | **Tissue** | **Disease** | **Carbohydrate** | **ST** | **GKI** | **GTR** | **MURI** | **MUTS** | **RECP** | **XPT** | **ATOB** | **CC_slv_** | **CC_dlv_** |
| --- | --- | --- | --- | --- | --- | --- | --- | --- | --- | --- | --- | --- | --- | --- | --- |
| B230 | emm23 | Mumbai | Throat | Carrier | G | 107 | 3 | 3 | 4 | 1 | 8 | 7 | 2 | 84 | 107 |
| C112 | stc1400 | Chennai | Throat | carrier | G | 66 | 3 | 3 | 4 | 2 | 9 | 22 | 2 | 66 | 66 |
| 488 | stc1400 | Mumbai | Skin | Pyoderma | C | 88 | 3 | 3 | 4 | 1 | 5 | 2 | 2 | 88 | 107 |
| 489 | stc1400 | Mumbai | Throat | Pharyngitis | C | 90 | 3 | 3 | 4 | 1 | 5 | 7 | 1 | 90 | 107 |
| C74 | stc2sk | Chennai | Skin | Pyoderma | G | 113 | 3 | 3 | 4 | 1 | 16 | 15 | 2 | 113 | 107 |
| B81 | stc36 | Mumbai | Throat | Carrier | G | 15 | 3 | 3 | 2 | 2 | 9 | 8 | 2 | 15 | 66 |
| B109 | stc36 | Mumbai | Throat | Carrier | G | 44 | 2 | 2 | 4 | 2 | 3 | 7 | 1 | 44 | 44 |
| B122 | stc36 | Mumbai | Throat | Carrier | G | 44 | 2 | 2 | 4 | 2 | 3 | 7 | 1 | 44 | 44 |
| B72 | stc36 | Mumbai | Throat | Carrier | G | 44 | 2 | 2 | 4 | 2 | 3 | 7 | 1 | 44 | 44 |
| B77 | stc36 | Mumbai | Throat | Carrier | G | 44 | 2 | 2 | 4 | 2 | 3 | 7 | 1 | 44 | 44 |
| C103 | stc36 | Chennai | Throat | carrier | G | 44 | 2 | 2 | 4 | 2 | 3 | 7 | 1 | 44 | 44 |
| C104 | stc36 | Chennai | Throat | carrier | G | 44 | 2 | 2 | 4 | 2 | 3 | 7 | 1 | 44 | 44 |
| C108 | stc36 | Chennai | Throat | carrier | G | 44 | 2 | 2 | 4 | 2 | 3 | 7 | 1 | 44 | 44 |
| C128 | stc36 | Chennai | Throat | carrier | G | 44 | 2 | 2 | 4 | 2 | 3 | 7 | 1 | 44 | 44 |
| C99 | stc36 | Chennai | Throat | carrier | G | 44 | 2 | 2 | 4 | 2 | 3 | 7 | 1 | 44 | 44 |
| C101 | stc36 | Chennai | Throat | Pharyngitis | G | 86 | 2 | 2 | 4 | 2 | 3 | 7 | 17 | 44 | 44 |
| B123 | stc36 | Mumbai | Throat | Pharyngitis | G | 96 | 2 | 2 | 4 | 12 | 3 | 7 | 1 | 44 | 44 |
| B203 | stc36 | Mumbai | Throat | Pharyngitis | C | 97 | 4 | 3 | 1 | 8 | 8 | 26 | 2 | 97 | 97 |
| B100 | stc36 | Mumbai | Throat | Pharyngitis | G | 98 | 2 | 2 | 5 | 2 | 3 | 7 | 1 | 44 | 44 |
| B272 | stC36 | Mumbai | Throat | Carrier | C | 109 | 2 | 4 | 4 | 16 | 24 | 29 | 2 | 109 | s |
| B71 | stc36 | Mumbai | Throat | Pharyngitis | G | 116 | 2 | 2 | 4 | 2 | 3 | 7 | 14 | 44 | 44 |
| C122 | stc5345 | Chennai | Throat | carrier | G | 107 | 3 | 3 | 4 | 1 | 8 | 7 | 2 | 84 | 107 |
| C125 | stc5345 | Chennai | Throat | carrier | G | 107 | 3 | 3 | 4 | 1 | 8 | 7 | 2 | 84 | 107 |
| C126 | stc5345 | Chennai | Throat | carrier | G | 107 | 3 | 3 | 4 | 1 | 8 | 7 | 2 | 84 | 107 |
| C137 | stc5345 | Chennai | Throat | carrier | G | 107 | 3 | 3 | 4 | 1 | 8 | 7 | 2 | 84 | 107 |
| C138 | stc5345 | Chennai | Throat | carrier | G | 107 | 3 | 3 | 4 | 1 | 8 | 7 | 2 | 84 | 107 |
| C143 | stc5345 | Chennai | Throat | carrier | G | 107 | 3 | 3 | 4 | 1 | 8 | 7 | 2 | 84 | 107 |
| 490 | stc6979 | Mumbai | Throat | Carrier | C | 107 | 3 | 3 | 4 | 1 | 8 | 7 | 2 | 84 | 107 |
| C105 | stc74a | Chennai | Throat | carrier | G | 29 | 3 | 2 | 4 | 2 | 7 | 1 | 3 | 29 | 29 |
| B180 | stC74a | Mumbai | Throat | Carrier | G | 83 | 3 | 10 | 4 | 13 | 8 | 7 | 2 | 83 | 107 |
| B242 | stc74a | Mumbai | Throat | Carrier | G | 83 | 3 | 10 | 4 | 13 | 8 | 7 | 2 | 83 | 107 |
| 491 | stcQ343 | Mumbai | Skin | Pyoderma | C | 91 | 2 | 12 | 4 | 2 | 4 | 4 | 2 | 91 | s |
| B51 | stg10 | Mumbai | Throat | Pharyngitis | G | 15 | 3 | 3 | 2 | 2 | 9 | 8 | 2 | 15 | 66 |
| B52 | stg10 | Mumbai | Throat | Carrier | G | 15 | 3 | 3 | 2 | 2 | 9 | 8 | 2 | 15 | 66 |
| B56 | stg10 | Mumbai | Throat | Carrier | G | 15 | 3 | 3 | 2 | 2 | 9 | 8 | 2 | 15 | 66 |
| C109 | stg166b | Chennai | Throat | carrier | G | 118 | 3 | 2 | 4 | 2 | 7 | 23 | 7 | 118 | 29 |
| C70 | stg1750 | Chennai | Throat | Tonsillitis | n.d. | 110 | 3 | 3 | 4 | 1 | 11 | 24 | 2 | 110 | 107 |
| C77 | stg1750 | Chennai | Skin | Impetigo | G | 110 | 3 | 3 | 4 | 1 | 11 | 24 | 2 | 110 | 107 |
| C80 | stg1750 | Chennai | Throat | Tonsillitis | n.d. | 110 | 3 | 3 | 4 | 1 | 11 | 24 | 2 | 110 | 107 |
| B134 | stg2078 | Mumbai | Throat | Carrier | G | 98 | 2 | 2 | 5 | 2 | 3 | 7 | 1 | 44 | 44 |
| B136 | stg2078 | Mumbai | Throat | Carrier | G | 98 | 2 | 2 | 5 | 2 | 3 | 7 | 1 | 44 | 44 |
| B244 | stG211 | Mumbai | Throat | Carrier | G | 108 | 4 | 3 | 1 | 8 | 25 | 26 | 2 | 97 | 97 |
| B113 | stg245 | Mumbai | Throat | Carrier | G | 15 | 3 | 3 | 2 | 2 | 9 | 8 | 2 | 15 | 66 |
| B114 | stg245 | Mumbai | Throat | Carrier | G | 15 | 3 | 3 | 2 | 2 | 9 | 8 | 2 | 15 | 66 |
| B158 | stg245 | Mumbai | Throat | Pharyngitis | G | 15 | 3 | 3 | 2 | 2 | 9 | 8 | 2 | 15 | 66 |
| B159 | stG245 | Mumbai | Throat | Carrier | G | 15 | 3 | 3 | 2 | 2 | 9 | 8 | 2 | 15 | 66 |
| B189 | stg245 | Mumbai | Throat | Carrier | G | 15 | 3 | 3 | 2 | 2 | 9 | 8 | 2 | 15 | 66 |
| B193 | stg245 | Mumbai | Throat | Pharyngitis | G | 15 | 3 | 3 | 2 | 2 | 9 | 8 | 2 | 15 | 66 |
| C117 | stg245 | Chennai | Throat | carrier | G | 29 | 3 | 2 | 4 | 2 | 7 | 1 | 3 | 29 | 29 |
| B63 | stg245 | Mumbai | Throat | Carrier | G | 44 | 2 | 2 | 4 | 2 | 3 | 7 | 1 | 44 | 44 |
| B92 | stg245 | Mumbai | Throat | Carrier | G | 81 | 3 | 2 | 4 | 1 | 5 | 7 | 2 | 81 | 107 |
| C107 | stg245 | Chennai | Throat | carrier | G | 81 | 3 | 2 | 4 | 1 | 5 | 7 | 2 | 81 | 107 |
| C115 | stg245 | Chennai | Throat | carrier | G | 81 | 3 | 2 | 4 | 1 | 5 | 7 | 2 | 81 | 107 |
| C132 | stg245 | Chennai | Throat | carrier | G | 81 | 3 | 2 | 4 | 1 | 5 | 7 | 2 | 81 | 107 |
| C136 | stg245 | Chennai | Unknown | Unknown | n.d. | 81 | 3 | 2 | 4 | 1 | 5 | 7 | 2 | 81 | 107 |
| C142 | stg245 | Chennai | Throat | carrier | G | 81 | 3 | 2 | 4 | 1 | 5 | 7 | 2 | 81 | 107 |
| C61 | stg245 | Chennai | Throat | Pharyngitis | G | 81 | 3 | 2 | 4 | 1 | 5 | 7 | 2 | 81 | 107 |
| B177 | stg245 | Mumbai | Throat | Carrier | G | 82 | 3 | 2 | 4 | 2 | 5 | 10 | 2 | 70 | 107 |
| B179 | stg245 | Mumbai | Throat | Carrier | G | 83 | 3 | 10 | 4 | 13 | 8 | 7 | 2 | 83 | 107 |
| C64 | stg245 | Chennai | Throat | Pharyngitis | n.d. | 110 | 3 | 3 | 4 | 1 | 11 | 24 | 2 | 110 | 107 |
| C75 | stg2574 | Chennai | Skin | Pyoderma | G | 113 | 3 | 3 | 4 | 1 | 16 | 15 | 2 | 113 | 107 |
| B204 | stG4222 | Mumbai | Throat | Pharyngitis | G | 105 | 3 | 3 | 9 | 14 | 8 | 28 | 2 | 105 | 107 |
| B225 | stg4222 | Mumbai | Throat | Carrier | C | 106 | 3 | 3 | 9 | 1 | 8 | 7 | 2 | 84 | 107 |
| B153 | stg480 | Mumbai | Throat | Carrier | G | 34 | 3 | 7 | 4 | 1 | 14 | 15 | 10 | 34 | 34 |
| B68 | stg480 | Mumbai | Throat | Pharyngitis | C | 34 | 3 | 7 | 4 | 1 | 14 | 15 | 10 | 34 | 34 |
| B160 | stg480 | Mumbai | Throat | Pharyngitis | C | 44 | 2 | 2 | 4 | 2 | 3 | 7 | 1 | 44 | 44 |
| B165 | stg480 | Mumbai | Throat | Pharyngitis | G | 98 | 2 | 2 | 5 | 2 | 3 | 7 | 1 | 44 | 44 |
| B231 | stg480 | Mumbai | Throat | Carrier | G | 98 | 2 | 2 | 5 | 2 | 3 | 7 | 1 | 44 | 44 |
| B232 | stg480 | Mumbai | Throat | Carrier | G | 98 | 2 | 2 | 5 | 2 | 3 | 7 | 1 | 44 | 44 |
| B82 | stg480 | Mumbai | Throat | Carrier | G | 98 | 2 | 2 | 5 | 2 | 3 | 7 | 1 | 44 | 44 |
| B112 | stg480 | Mumbai | Throat | Carrier | G | 99 | 2 | 2 | 5 | 2 | 3 | 7 | 2 | 44 | 44 |
| C124 | stg480 | Chennai | Throat | carrier | G | 121 | 2 | 3 | 4 | 2 | 9 | 22 | 2 | 66 | 66 |
| B11 | stg4831 | Mumbai | Throat | Pharyngitis | G | 84 | 6 | 3 | 9 | 1 | 8 | 3 | 2 | 84 | 107 |
| B12 | stg4831 | Mumbai | Throat | Pharyngitis | G | 84 | 6 | 3 | 9 | 1 | 8 | 3 | 2 | 84 | 107 |
| B13 | stg4831 | Mumbai | Throat | Carrier | G | 84 | 6 | 3 | 9 | 1 | 8 | 3 | 2 | 84 | 107 |
| B18 | stg4831 | Mumbai | Throat | Carrier | G | 84 | 6 | 3 | 9 | 1 | 8 | 3 | 2 | 84 | 107 |
| B19 | stg4831 | Mumbai | Throat | Carrier | G | 84 | 6 | 3 | 9 | 1 | 8 | 3 | 2 | 84 | 107 |
| B196 | stg4831 | Mumbai | Throat | Carrier | G | 84 | 6 | 3 | 9 | 1 | 8 | 3 | 2 | 84 | 107 |
| B197 | stg4831 | Mumbai | Throat | Carrier | G | 84 | 6 | 3 | 9 | 1 | 8 | 3 | 2 | 84 | 107 |
| B20 | stg4831 | Mumbai | Throat | Carrier | G | 84 | 6 | 3 | 9 | 1 | 8 | 3 | 2 | 84 | 107 |
| B21 | stg4831 | Mumbai | Throat | Pharyngitis | G | 84 | 6 | 3 | 9 | 1 | 8 | 3 | 2 | 84 | 107 |
| B219 | stg4831 | Mumbai | Throat | Carrier | G | 84 | 6 | 3 | 9 | 1 | 8 | 3 | 2 | 84 | 107 |
| B22 | stg4831 | Mumbai | Throat | Pharyngitis | G | 84 | 6 | 3 | 9 | 1 | 8 | 3 | 2 | 84 | 107 |
| B23 | stg4831 | Mumbai | Throat | Carrier | G | 84 | 6 | 3 | 9 | 1 | 8 | 3 | 2 | 84 | 107 |
| B24 | stg4831 | Mumbai | Throat | Pharyngitis | G | 84 | 6 | 3 | 9 | 1 | 8 | 3 | 2 | 84 | 107 |
| B248 | stg4831 | Mumbai | Throat | Pharyngitis | G | 84 | 6 | 3 | 9 | 1 | 8 | 3 | 2 | 84 | 107 |
| B25 | stg4831 | Mumbai | Throat | Carrier | G | 84 | 6 | 3 | 9 | 1 | 8 | 3 | 2 | 84 | 107 |
| B252 | stg4831 | Mumbai | Throat | Carrier | G | 84 | 6 | 3 | 9 | 1 | 8 | 3 | 2 | 84 | 107 |
| B26 | stg4831 | Mumbai | Throat | Pharyngitis | G | 84 | 6 | 3 | 9 | 1 | 8 | 3 | 2 | 84 | 107 |
| B27 | stg4831 | Mumbai | Throat | Pharyngitis | G | 84 | 6 | 3 | 9 | 1 | 8 | 3 | 2 | 84 | 107 |
| B28 | stg4831 | Mumbai | Throat | Carrier | G | 84 | 6 | 3 | 9 | 1 | 8 | 3 | 2 | 84 | 107 |
| B29 | stg4831 | Mumbai | Throat | Pharyngitis | G | 84 | 6 | 3 | 9 | 1 | 8 | 3 | 2 | 84 | 107 |
| B30 | stg4831 | Mumbai | Throat | Pharyngitis | G | 84 | 6 | 3 | 9 | 1 | 8 | 3 | 2 | 84 | 107 |
| B33 | stg4831 | Mumbai | Throat | Pharyngitis | G | 84 | 6 | 3 | 9 | 1 | 8 | 3 | 2 | 84 | 107 |
| B34 | stg4831 | Mumbai | Throat | Pharyngitis | G | 84 | 6 | 3 | 9 | 1 | 8 | 3 | 2 | 84 | 107 |
| B35 | stg4831 | Mumbai | Throat | Pharyngitis | G | 84 | 6 | 3 | 9 | 1 | 8 | 3 | 2 | 84 | 107 |
| B36 | stg4831 | Mumbai | Throat | Carrier | G | 84 | 6 | 3 | 9 | 1 | 8 | 3 | 2 | 84 | 107 |
| B37 | stg4831 | Mumbai | Throat | Pharyngitis | G | 84 | 6 | 3 | 9 | 1 | 8 | 3 | 2 | 84 | 107 |
| B38 | stg4831 | Mumbai | Throat | Carrier | G | 84 | 6 | 3 | 9 | 1 | 8 | 3 | 2 | 84 | 107 |
| B39 | stg4831 | Mumbai | Throat | Carrier | G | 84 | 6 | 3 | 9 | 1 | 8 | 3 | 2 | 84 | 107 |
| B40 | stg4831 | Mumbai | Throat | Pharyngitis | G | 84 | 6 | 3 | 9 | 1 | 8 | 3 | 2 | 84 | 107 |
| B41 | stg4831 | Mumbai | Throat | Pharyngitis | G | 84 | 6 | 3 | 9 | 1 | 8 | 3 | 2 | 84 | 107 |
| B42 | stg4831 | Mumbai | Throat | Carrier | G | 84 | 6 | 3 | 9 | 1 | 8 | 3 | 2 | 84 | 107 |
| B43 | stg4831 | Mumbai | Throat | Pharyngitis | G | 84 | 6 | 3 | 9 | 1 | 8 | 3 | 2 | 84 | 107 |
| B44 | stg4831 | Mumbai | Throat | Carrier | G | 84 | 6 | 3 | 9 | 1 | 8 | 3 | 2 | 84 | 107 |
| B48 | stg4831 | Mumbai | Throat | Carrier | G | 84 | 6 | 3 | 9 | 1 | 8 | 3 | 2 | 84 | 107 |
| B49 | stg4831 | Mumbai | Throat | Pharyngitis | G | 84 | 6 | 3 | 9 | 1 | 8 | 3 | 2 | 84 | 107 |
| B5 | stg4831 | Mumbai | Throat | Carrier | G | 84 | 6 | 3 | 9 | 1 | 8 | 3 | 2 | 84 | 107 |
| B50 | stg4831 | Mumbai | Throat | Pharyngitis | G | 84 | 6 | 3 | 9 | 1 | 8 | 3 | 2 | 84 | 107 |
| B55 | stg4831 | Mumbai | Throat | Pharyngitis | G | 84 | 6 | 3 | 9 | 1 | 8 | 3 | 2 | 84 | 107 |
| B6 | stg4831 | Mumbai | Throat | Pharyngitis | G | 84 | 6 | 3 | 9 | 1 | 8 | 3 | 2 | 84 | 107 |
| B60 | stg4831 | Mumbai | Throat | Pharyngitis | G | 84 | 6 | 3 | 9 | 1 | 8 | 3 | 2 | 84 | 107 |
| B7 | stg4831 | Mumbai | Throat | Carrier | G | 84 | 6 | 3 | 9 | 1 | 8 | 3 | 2 | 84 | 107 |
| B8 | stg4831 | Mumbai | Throat | Carrier | G | 84 | 6 | 3 | 9 | 1 | 8 | 3 | 2 | 84 | 107 |
| B9 | stg4831 | Mumbai | Throat | Pharyngitis | G | 84 | 6 | 3 | 9 | 1 | 8 | 3 | 2 | 84 | 107 |
| B17 | stg4831 | Mumbai | Throat | Carrier | G | 85 | 6 | 3 | 9 | 11 | 22 | 3 | 2 | 85 | 107 |
| B220 | stg4831 | Mumbai | Throat | Carrier | G | 87 | 3 | 3 | 9 | 1 | 8 | 3 | 2 | 84 | 107 |
| B199 | stG4831 | Mumbai | Throat | Carrier | G | 102 | 6 | 3 | 5 | 1 | 9 | 30 | 2 | 102 | 89 |
| C100 | stg485 | Chennai | Throat | carrier | G | 100 | 4 | 2 | 4 | 2 | 9 | 22 | 16 | 100 | s |
| C111 | stg4974 | Chennai | Throat | carrier | G | 119 | 4 | 3 | 4 | 1 | 21 | 3 | 2 | 119 | s |
| C133 | stg4974 | Chennai | Throat | carrier | G | 119 | 4 | 3 | 4 | 1 | 21 | 3 | 2 | 119 | s |
| C141 | stg4974 | Chennai | Throat | carrier | G | 119 | 4 | 3 | 4 | 1 | 21 | 3 | 2 | 119 | s |
| 497 | stg6 | Mumbai | Skin | Pyoderma | G | 44 | 2 | 2 | 4 | 2 | 3 | 7 | 1 | 44 | 44 |
| B102 | stg6 | Mumbai | Throat | Carrier | G | 44 | 2 | 2 | 4 | 2 | 3 | 7 | 1 | 44 | 44 |
| B84 | stg6 | Mumbai | Throat | Pharyngitis | G | 44 | 2 | 2 | 4 | 2 | 3 | 7 | 1 | 44 | 44 |
| B93 | stg6 | Mumbai | Throat | Pharyngitis | C | 44 | 2 | 2 | 4 | 2 | 3 | 7 | 1 | 44 | 44 |
| B110 | stg6 | Mumbai | Throat | Carrier | C | 81 | 3 | 2 | 4 | 1 | 5 | 7 | 2 | 81 | 107 |
| 496 | stg6 | Mumbai | Skin | Pyoderma | G | 117 | 15 | 2 | 4 | 2 | 3 | 7 | 1 | 44 | 44 |
| 498 | stg6 | Mumbai | Skin | Pyoderma | G | 122 | 16 | 2 | 4 | 1 | 3 | 7 | 15 | 122 | 44 |
| 499 | stg6 | Mumbai | Skin | Pyoderma | G | 123 | 16 | 2 | 4 | 2 | 3 | 7 | 1 | 44 | 44 |
| 500 | stg6 | Mumbai | Skin | Pyoderma | G | 123 | 16 | 2 | 4 | 2 | 3 | 7 | 1 | 44 | 44 |
| 501 | stg6 | Mumbai | Throat | Pharyngitis | G | 124 | 3 | 2 | 4 | 1 | 4 | 1 | 2 | 124 | 107 |
| 502 | stg6 | Mumbai | Throat | Carrier | G | 124 | 3 | 2 | 4 | 1 | 4 | 1 | 2 | 124 | 107 |
| C66 | stg643 | Chennai | Throat | Tonsillitis | G | 112 | 2 | 2 | 4 | 2 | 3 | 7 | 7 | 44 | 44 |
| C67 | stg643 | Chennai | Throat | Tonsillitis | G | 112 | 2 | 2 | 4 | 2 | 3 | 7 | 7 | 44 | 44 |
| C123 | stg643 | Chennai | Throat | carrier | G | 120 | 2 | 2 | 12 | 2 | 3 | 7 | 7 | 44 | 44 |
| B124 | stg652 | Mumbai | Throat | Pharyngitis | G | 70 | 3 | 10 | 4 | 1 | 5 | 10 | 2 | 70 | 107 |
| B69 | stg652 | Mumbai | Throat | Pharyngitis | G | 70 | 3 | 10 | 4 | 1 | 5 | 10 | 2 | 70 | 107 |
| C119 | stg652 | Chennai | Throat | carrier | G | 70 | 3 | 10 | 4 | 1 | 5 | 10 | 2 | 70 | 107 |
| C88 | stg652 | Chennai | Throat | Pharyngitis | n.d. | 114 | 3 | 10 | 4 | 2 | 5 | 10 | 2 | 70 | 107 |
| C95 | stg652 | Chennai | Skin | wound swab | n.d. | 115 | 3 | 2 | 1 | 2 | 5 | 10 | 2 | 70 | 107 |
| B10 | stg653 | Mumbai | Throat | Pharyngitis | G | 95 | 2 | 2 | 5 | 2 | 3 | 7 | 13 | 44 | 44 |
| B127 | stg653 | Mumbai | Throat | Carrier | G | 95 | 2 | 2 | 5 | 2 | 3 | 7 | 13 | 44 | 44 |
| B151 | stg653 | Mumbai | Throat | Carrier | C | 95 | 2 | 2 | 5 | 2 | 3 | 7 | 13 | 44 | 44 |
| C116 | stg653 | Chennai | Throat | carrier | G | 95 | 2 | 2 | 5 | 2 | 3 | 7 | 13 | 44 | 44 |
| C69 | stg653 | Chennai | Throat | Pharyngitis | n.d. | 95 | 2 | 2 | 5 | 2 | 3 | 7 | 13 | 44 | 44 |
| B202 | stG653 | Mumbai | Throat | Carrier | G | 104 | 5 | 3 | 5 | 1 | 6 | 2 | 1 | 104 | s |
| B126 | stg6792 | Mumbai | Throat | Pharyngitis | G | 66 | 3 | 3 | 4 | 2 | 9 | 22 | 2 | 66 | 66 |
| B150 | stg6792 | Mumbai | Throat | Carrier | C | 66 | 3 | 3 | 4 | 2 | 9 | 22 | 2 | 66 | 66 |
| B66 | stg6792 | Mumbai | Throat | Carrier | G | 66 | 3 | 3 | 4 | 2 | 9 | 22 | 2 | 66 | 66 |
| C129 | stg6792 | Chennai | Throat | carrier | G | 66 | 3 | 3 | 4 | 2 | 9 | 22 | 2 | 66 | 66 |
| C63 | stg6792 | Chennai | Throat | Tonsillitis | n.d. | 66 | 3 | 3 | 4 | 2 | 9 | 22 | 2 | 66 | 66 |
| C98 | stg6792 | Chennai | Cellular fluid | cellular fluid | n.d. | 66 | 3 | 3 | 4 | 2 | 9 | 22 | 2 | 66 | 66 |
| B67 | stg6792 | Mumbai | Throat | Carrier | G | 95 | 2 | 2 | 5 | 2 | 3 | 7 | 13 | 44 | 44 |
| C65 | stg6792 | Chennai | Throat | Tonsillitis | G | 111 | 3 | 3 | 4 | 2 | 9 | 25 | 2 | 66 | 66 |
| C82 | stg6792 | Chennai | Throat | Pharyngitis | n.d. | 111 | 3 | 3 | 4 | 2 | 9 | 25 | 2 | 66 | 66 |
| C86 | stg6792 | Chennai | Throat | Tonsillitis | G | 111 | 3 | 3 | 4 | 2 | 9 | 25 | 2 | 66 | 66 |
| C90 | stg6792 | Chennai | Throat | Pharyngitis | n.d. | 111 | 3 | 3 | 4 | 2 | 9 | 25 | 2 | 66 | 66 |
| C91 | stg6792 | Chennai | Throat | Pharyngitis | n.d. | 111 | 3 | 3 | 4 | 2 | 9 | 25 | 2 | 66 | 66 |
| C121 | stg840 | Chennai | Throat | carrier | G | 34 | 3 | 7 | 4 | 1 | 14 | 15 | 10 | 34 | 34 |
| B255 | stg866 | Mumbai | Throat | Carrier | G | 15 | 3 | 3 | 2 | 2 | 9 | 8 | 2 | 15 | 66 |
| B31 | stg866 | Mumbai | Throat | Pharyngitis | G | 89 | 13 | 3 | 5 | 1 | 9 | 26 | 2 | 89 | 89 |
| B32 | stg866 | Mumbai | Throat | Pharyngitis | G | 89 | 13 | 3 | 5 | 1 | 9 | 26 | 2 | 89 | 89 |
| B45 | stg866 | Mumbai | Throat | Pharyngitis | G | 89 | 13 | 3 | 5 | 1 | 9 | 26 | 2 | 89 | 89 |
| B46 | stg866 | Mumbai | Throat | Pharyngitis | G | 89 | 13 | 3 | 5 | 1 | 9 | 26 | 2 | 89 | 89 |
| B47 | stg866 | Mumbai | Throat | Pharyngitis | G | 89 | 13 | 3 | 5 | 1 | 9 | 26 | 2 | 89 | 89 |
| B85 | stg866 | Mumbai | Throat | Carrier | G | 89 | 13 | 3 | 5 | 1 | 9 | 26 | 2 | 89 | 89 |
| C79 | stg866 | Chennai | Throat | Tonsillitis | G | 89 | 13 | 3 | 5 | 1 | 9 | 26 | 2 | 89 | 89 |
| C81 | stg866 | Chennai | Throat | Tonsillitis | G | 89 | 13 | 3 | 5 | 1 | 9 | 26 | 2 | 89 | 89 |
| C85 | stg866 | Chennai | Throat | Tonsillitis | G | 89 | 13 | 3 | 5 | 1 | 9 | 26 | 2 | 89 | 89 |
| B210 | stg866 | Mumbai | Throat | Carrier | G | 92 | 13 | 3 | 14 | 1 | 22 | 26 | 14 | 92 | s |
| B212 | stg866 | Mumbai | Throat | Carrier | G | 93 | 13 | 3 | 5 | 1 | 8 | 26 | 2 | 89 | 89 |
| B111 | stg866 | Mumbai | Throat | Carrier | G | 126 | 13 | 3 | 15 | 1 | 9 | 26 | 2 | 89 | 89 |
| B184 | stgL265 | Mumbai | Throat | Carrier | G | 15 | 3 | 3 | 2 | 2 | 9 | 8 | 2 | 15 | 66 |
| B215 | stGL265 | Mumbai | Throat | Pharyngitis | G | 44 | 2 | 2 | 4 | 2 | 3 | 7 | 1 | 44 | 44 |
| B217 | stgL265 | Mumbai | Throat | Carrier | G | 101 | 2 | 2 | 4 | 15 | 3 | 7 | 1 | 44 | 44 |
| B201 | stgLP2 | Mumbai | Throat | Carrier | G | 103 | 2 | 2 | 4 | 2 | 3 | 7 | 2 | 44 | 44 |
| B152 | stgm22 | Mumbai | Throat | Carrier | C | 34 | 3 | 7 | 4 | 1 | 14 | 15 | 10 | 34 | 34 |
| B65 | stgm22 | Mumbai | Throat | Carrier | G | 34 | 3 | 7 | 4 | 1 | 14 | 15 | 10 | 34 | 34 |
| B128 | stgm22 | Mumbai | Throat | Carrier | G | 94 | 3 | 7 | 13 | 1 | 14 | 15 | 10 | 34 | 34 |
| B129 | stgm22 | Mumbai | Throat | Carrier | G | 125 | 3 | 10 | 4 | 1 | 14 | 15 | 10 | 34 | 34 |
| **Strain** | **Emm-type** | **Source** | **Tissue** | **Disease** | **Carbohydrate** | **ST** | **GKI** | **GTR** | **MURI** | **MUTS** | **RECP** | **XPT** | **AtoB** | **CC_slv_** | **CC_dlv_** |
| B230 | emm23 | Mumbai | Throat | Carrier | G | 107 | 3 | 3 | 4 | 1 | 8 | 7 | 2 | 84 | 107 |
| C112 | stc1400 | Chennai | Throat | carrier | G | 66 | 3 | 3 | 4 | 2 | 9 | 22 | 2 | 66 | 66 |
| 488 | stc1400 | Mumbai | Skin | Pyoderma | C | 88 | 3 | 3 | 4 | 1 | 5 | 2 | 2 | 88 | 107 |
| 489 | stc1400 | Mumbai | Throat | Pharyngitis | C | 90 | 3 | 3 | 4 | 1 | 5 | 7 | 1 | 90 | 107 |
| C74 | stc2sk | Chennai | Skin | Pyoderma | G | 113 | 3 | 3 | 4 | 1 | 16 | 15 | 2 | 113 | 107 |
| B81 | stc36 | Mumbai | Throat | Carrier | G | 15 | 3 | 3 | 2 | 2 | 9 | 8 | 2 | 15 | 66 |
| B109 | stc36 | Mumbai | Throat | Carrier | G | 44 | 2 | 2 | 4 | 2 | 3 | 7 | 1 | 44 | 44 |
| B122 | stc36 | Mumbai | Throat | Carrier | G | 44 | 2 | 2 | 4 | 2 | 3 | 7 | 1 | 44 | 44 |
| B72 | stc36 | Mumbai | Throat | Carrier | G | 44 | 2 | 2 | 4 | 2 | 3 | 7 | 1 | 44 | 44 |
| B77 | stc36 | Mumbai | Throat | Carrier | G | 44 | 2 | 2 | 4 | 2 | 3 | 7 | 1 | 44 | 44 |
| C103 | stc36 | Chennai | Throat | carrier | G | 44 | 2 | 2 | 4 | 2 | 3 | 7 | 1 | 44 | 44 |
| C104 | stc36 | Chennai | Throat | carrier | G | 44 | 2 | 2 | 4 | 2 | 3 | 7 | 1 | 44 | 44 |
| C108 | stc36 | Chennai | Throat | carrier | G | 44 | 2 | 2 | 4 | 2 | 3 | 7 | 1 | 44 | 44 |
| C128 | stc36 | Chennai | Throat | carrier | G | 44 | 2 | 2 | 4 | 2 | 3 | 7 | 1 | 44 | 44 |
| C99 | stc36 | Chennai | Throat | carrier | G | 44 | 2 | 2 | 4 | 2 | 3 | 7 | 1 | 44 | 44 |
| C101 | stc36 | Chennai | Throat | Pharyngitis | G | 86 | 2 | 2 | 4 | 2 | 3 | 7 | 17 | 44 | 44 |
| B123 | stc36 | Mumbai | Throat | Pharyngitis | G | 96 | 2 | 2 | 4 | 12 | 3 | 7 | 1 | 44 | 44 |
| B203 | stc36 | Mumbai | Throat | Pharyngitis | C | 97 | 4 | 3 | 1 | 8 | 8 | 26 | 2 | 97 | 97 |
| B100 | stc36 | Mumbai | Throat | Pharyngitis | G | 98 | 2 | 2 | 5 | 2 | 3 | 7 | 1 | 44 | 44 |
| B272 | stC36 | Mumbai | Throat | Carrier | C | 109 | 2 | 4 | 4 | 16 | 24 | 29 | 2 | 109 | s |
| B71 | stc36 | Mumbai | Throat | Pharyngitis | G | 116 | 2 | 2 | 4 | 2 | 3 | 7 | 14 | 44 | 44 |
| C122 | stc5345 | Chennai | Throat | carrier | G | 107 | 3 | 3 | 4 | 1 | 8 | 7 | 2 | 84 | 107 |
| C125 | stc5345 | Chennai | Throat | carrier | G | 107 | 3 | 3 | 4 | 1 | 8 | 7 | 2 | 84 | 107 |
| C126 | stc5345 | Chennai | Throat | carrier | G | 107 | 3 | 3 | 4 | 1 | 8 | 7 | 2 | 84 | 107 |
| C137 | stc5345 | Chennai | Throat | carrier | G | 107 | 3 | 3 | 4 | 1 | 8 | 7 | 2 | 84 | 107 |
| C138 | stc5345 | Chennai | Throat | carrier | G | 107 | 3 | 3 | 4 | 1 | 8 | 7 | 2 | 84 | 107 |
| C143 | stc5345 | Chennai | Throat | carrier | G | 107 | 3 | 3 | 4 | 1 | 8 | 7 | 2 | 84 | 107 |
| 490 | stc6979 | Mumbai | Throat | Carrier | C | 107 | 3 | 3 | 4 | 1 | 8 | 7 | 2 | 84 | 107 |
| C105 | stc74a | Chennai | Throat | carrier | G | 29 | 3 | 2 | 4 | 2 | 7 | 1 | 3 | 29 | 29 |
| B180 | stC74a | Mumbai | Throat | Carrier | G | 83 | 3 | 10 | 4 | 13 | 8 | 7 | 2 | 83 | 107 |
| B242 | stc74a | Mumbai | Throat | Carrier | G | 83 | 3 | 10 | 4 | 13 | 8 | 7 | 2 | 83 | 107 |
| 491 | stcQ343 | Mumbai | Skin | Pyoderma | C | 91 | 2 | 12 | 4 | 2 | 4 | 4 | 2 | 91 | s |
| B51 | stg10 | Mumbai | Throat | Pharyngitis | G | 15 | 3 | 3 | 2 | 2 | 9 | 8 | 2 | 15 | 66 |
| B52 | stg10 | Mumbai | Throat | Carrier | G | 15 | 3 | 3 | 2 | 2 | 9 | 8 | 2 | 15 | 66 |
| B56 | stg10 | Mumbai | Throat | Carrier | G | 15 | 3 | 3 | 2 | 2 | 9 | 8 | 2 | 15 | 66 |
| C109 | stg166b | Chennai | Throat | carrier | G | 118 | 3 | 2 | 4 | 2 | 7 | 23 | 7 | 118 | 29 |
| C70 | stg1750 | Chennai | Throat | Tonsillitis | n.d. | 110 | 3 | 3 | 4 | 1 | 11 | 24 | 2 | 110 | 107 |
| C77 | stg1750 | Chennai | Skin | Impetigo | G | 110 | 3 | 3 | 4 | 1 | 11 | 24 | 2 | 110 | 107 |
| C80 | stg1750 | Chennai | Throat | Tonsillitis | n.d. | 110 | 3 | 3 | 4 | 1 | 11 | 24 | 2 | 110 | 107 |
| B134 | stg2078 | Mumbai | Throat | Carrier | G | 98 | 2 | 2 | 5 | 2 | 3 | 7 | 1 | 44 | 44 |
| B136 | stg2078 | Mumbai | Throat | Carrier | G | 98 | 2 | 2 | 5 | 2 | 3 | 7 | 1 | 44 | 44 |
| B244 | stG211 | Mumbai | Throat | Carrier | G | 108 | 4 | 3 | 1 | 8 | 25 | 26 | 2 | 97 | 97 |
| B113 | stg245 | Mumbai | Throat | Carrier | G | 15 | 3 | 3 | 2 | 2 | 9 | 8 | 2 | 15 | 66 |
| B114 | stg245 | Mumbai | Throat | Carrier | G | 15 | 3 | 3 | 2 | 2 | 9 | 8 | 2 | 15 | 66 |
| B158 | stg245 | Mumbai | Throat | Pharyngitis | G | 15 | 3 | 3 | 2 | 2 | 9 | 8 | 2 | 15 | 66 |
| B159 | stG245 | Mumbai | Throat | Carrier | G | 15 | 3 | 3 | 2 | 2 | 9 | 8 | 2 | 15 | 66 |
| B189 | stg245 | Mumbai | Throat | Carrier | G | 15 | 3 | 3 | 2 | 2 | 9 | 8 | 2 | 15 | 66 |
| B193 | stg245 | Mumbai | Throat | Pharyngitis | G | 15 | 3 | 3 | 2 | 2 | 9 | 8 | 2 | 15 | 66 |
| C117 | stg245 | Chennai | Throat | carrier | G | 29 | 3 | 2 | 4 | 2 | 7 | 1 | 3 | 29 | 29 |
| B63 | stg245 | Mumbai | Throat | Carrier | G | 44 | 2 | 2 | 4 | 2 | 3 | 7 | 1 | 44 | 44 |
| B92 | stg245 | Mumbai | Throat | Carrier | G | 81 | 3 | 2 | 4 | 1 | 5 | 7 | 2 | 81 | 107 |
| C107 | stg245 | Chennai | Throat | carrier | G | 81 | 3 | 2 | 4 | 1 | 5 | 7 | 2 | 81 | 107 |
| C115 | stg245 | Chennai | Throat | carrier | G | 81 | 3 | 2 | 4 | 1 | 5 | 7 | 2 | 81 | 107 |
| C132 | stg245 | Chennai | Throat | carrier | G | 81 | 3 | 2 | 4 | 1 | 5 | 7 | 2 | 81 | 107 |
| C136 | stg245 | Chennai | Unknown | Unknown | n.d. | 81 | 3 | 2 | 4 | 1 | 5 | 7 | 2 | 81 | 107 |
| C142 | stg245 | Chennai | Throat | carrier | G | 81 | 3 | 2 | 4 | 1 | 5 | 7 | 2 | 81 | 107 |
| C61 | stg245 | Chennai | Throat | Pharyngitis | G | 81 | 3 | 2 | 4 | 1 | 5 | 7 | 2 | 81 | 107 |
| B177 | stg245 | Mumbai | Throat | Carrier | G | 82 | 3 | 2 | 4 | 2 | 5 | 10 | 2 | 70 | 107 |
| B179 | stg245 | Mumbai | Throat | Carrier | G | 83 | 3 | 10 | 4 | 13 | 8 | 7 | 2 | 83 | 107 |
| C64 | stg245 | Chennai | Throat | Pharyngitis | n.d. | 110 | 3 | 3 | 4 | 1 | 11 | 24 | 2 | 110 | 107 |
| C75 | stg2574 | Chennai | Skin | Pyoderma | G | 113 | 3 | 3 | 4 | 1 | 16 | 15 | 2 | 113 | 107 |
| B204 | stG4222 | Mumbai | Throat | Pharyngitis | G | 105 | 3 | 3 | 9 | 14 | 8 | 28 | 2 | 105 | 107 |
| B225 | stg4222 | Mumbai | Throat | Carrier | C | 106 | 3 | 3 | 9 | 1 | 8 | 7 | 2 | 84 | 107 |
| B153 | stg480 | Mumbai | Throat | Carrier | G | 34 | 3 | 7 | 4 | 1 | 14 | 15 | 10 | 34 | 34 |
| B68 | stg480 | Mumbai | Throat | Pharyngitis | C | 34 | 3 | 7 | 4 | 1 | 14 | 15 | 10 | 34 | 34 |
| B160 | stg480 | Mumbai | Throat | Pharyngitis | C | 44 | 2 | 2 | 4 | 2 | 3 | 7 | 1 | 44 | 44 |
| B165 | stg480 | Mumbai | Throat | Pharyngitis | G | 98 | 2 | 2 | 5 | 2 | 3 | 7 | 1 | 44 | 44 |
| B231 | stg480 | Mumbai | Throat | Carrier | G | 98 | 2 | 2 | 5 | 2 | 3 | 7 | 1 | 44 | 44 |
| B232 | stg480 | Mumbai | Throat | Carrier | G | 98 | 2 | 2 | 5 | 2 | 3 | 7 | 1 | 44 | 44 |
| B82 | stg480 | Mumbai | Throat | Carrier | G | 98 | 2 | 2 | 5 | 2 | 3 | 7 | 1 | 44 | 44 |
| B112 | stg480 | Mumbai | Throat | Carrier | G | 99 | 2 | 2 | 5 | 2 | 3 | 7 | 2 | 44 | 44 |
| C124 | stg480 | Chennai | Throat | carrier | G | 121 | 2 | 3 | 4 | 2 | 9 | 22 | 2 | 66 | 66 |
| B11 | stg4831 | Mumbai | Throat | Pharyngitis | G | 84 | 6 | 3 | 9 | 1 | 8 | 3 | 2 | 84 | 107 |
| B12 | stg4831 | Mumbai | Throat | Pharyngitis | G | 84 | 6 | 3 | 9 | 1 | 8 | 3 | 2 | 84 | 107 |
| B13 | stg4831 | Mumbai | Throat | Carrier | G | 84 | 6 | 3 | 9 | 1 | 8 | 3 | 2 | 84 | 107 |
| B18 | stg4831 | Mumbai | Throat | Carrier | G | 84 | 6 | 3 | 9 | 1 | 8 | 3 | 2 | 84 | 107 |
| B19 | stg4831 | Mumbai | Throat | Carrier | G | 84 | 6 | 3 | 9 | 1 | 8 | 3 | 2 | 84 | 107 |
| B196 | stg4831 | Mumbai | Throat | Carrier | G | 84 | 6 | 3 | 9 | 1 | 8 | 3 | 2 | 84 | 107 |
| B197 | stg4831 | Mumbai | Throat | Carrier | G | 84 | 6 | 3 | 9 | 1 | 8 | 3 | 2 | 84 | 107 |
| B20 | stg4831 | Mumbai | Throat | Carrier | G | 84 | 6 | 3 | 9 | 1 | 8 | 3 | 2 | 84 | 107 |
| B21 | stg4831 | Mumbai | Throat | Pharyngitis | G | 84 | 6 | 3 | 9 | 1 | 8 | 3 | 2 | 84 | 107 |
| B219 | stg4831 | Mumbai | Throat | Carrier | G | 84 | 6 | 3 | 9 | 1 | 8 | 3 | 2 | 84 | 107 |
| B22 | stg4831 | Mumbai | Throat | Pharyngitis | G | 84 | 6 | 3 | 9 | 1 | 8 | 3 | 2 | 84 | 107 |
| B23 | stg4831 | Mumbai | Throat | Carrier | G | 84 | 6 | 3 | 9 | 1 | 8 | 3 | 2 | 84 | 107 |
| B24 | stg4831 | Mumbai | Throat | Pharyngitis | G | 84 | 6 | 3 | 9 | 1 | 8 | 3 | 2 | 84 | 107 |
| B248 | stg4831 | Mumbai | Throat | Pharyngitis | G | 84 | 6 | 3 | 9 | 1 | 8 | 3 | 2 | 84 | 107 |
| B25 | stg4831 | Mumbai | Throat | Carrier | G | 84 | 6 | 3 | 9 | 1 | 8 | 3 | 2 | 84 | 107 |
| B252 | stg4831 | Mumbai | Throat | Carrier | G | 84 | 6 | 3 | 9 | 1 | 8 | 3 | 2 | 84 | 107 |
| B26 | stg4831 | Mumbai | Throat | Pharyngitis | G | 84 | 6 | 3 | 9 | 1 | 8 | 3 | 2 | 84 | 107 |
| B27 | stg4831 | Mumbai | Throat | Pharyngitis | G | 84 | 6 | 3 | 9 | 1 | 8 | 3 | 2 | 84 | 107 |
| B28 | stg4831 | Mumbai | Throat | Carrier | G | 84 | 6 | 3 | 9 | 1 | 8 | 3 | 2 | 84 | 107 |
| B29 | stg4831 | Mumbai | Throat | Pharyngitis | G | 84 | 6 | 3 | 9 | 1 | 8 | 3 | 2 | 84 | 107 |
| B30 | stg4831 | Mumbai | Throat | Pharyngitis | G | 84 | 6 | 3 | 9 | 1 | 8 | 3 | 2 | 84 | 107 |
| B33 | stg4831 | Mumbai | Throat | Pharyngitis | G | 84 | 6 | 3 | 9 | 1 | 8 | 3 | 2 | 84 | 107 |
| B34 | stg4831 | Mumbai | Throat | Pharyngitis | G | 84 | 6 | 3 | 9 | 1 | 8 | 3 | 2 | 84 | 107 |
| B35 | stg4831 | Mumbai | Throat | Pharyngitis | G | 84 | 6 | 3 | 9 | 1 | 8 | 3 | 2 | 84 | 107 |
| B36 | stg4831 | Mumbai | Throat | Carrier | G | 84 | 6 | 3 | 9 | 1 | 8 | 3 | 2 | 84 | 107 |
| B37 | stg4831 | Mumbai | Throat | Pharyngitis | G | 84 | 6 | 3 | 9 | 1 | 8 | 3 | 2 | 84 | 107 |
| B38 | stg4831 | Mumbai | Throat | Carrier | G | 84 | 6 | 3 | 9 | 1 | 8 | 3 | 2 | 84 | 107 |
| B39 | stg4831 | Mumbai | Throat | Carrier | G | 84 | 6 | 3 | 9 | 1 | 8 | 3 | 2 | 84 | 107 |
| B40 | stg4831 | Mumbai | Throat | Pharyngitis | G | 84 | 6 | 3 | 9 | 1 | 8 | 3 | 2 | 84 | 107 |
| B41 | stg4831 | Mumbai | Throat | Pharyngitis | G | 84 | 6 | 3 | 9 | 1 | 8 | 3 | 2 | 84 | 107 |
| B42 | stg4831 | Mumbai | Throat | Carrier | G | 84 | 6 | 3 | 9 | 1 | 8 | 3 | 2 | 84 | 107 |
| B43 | stg4831 | Mumbai | Throat | Pharyngitis | G | 84 | 6 | 3 | 9 | 1 | 8 | 3 | 2 | 84 | 107 |
| B44 | stg4831 | Mumbai | Throat | Carrier | G | 84 | 6 | 3 | 9 | 1 | 8 | 3 | 2 | 84 | 107 |
| B48 | stg4831 | Mumbai | Throat | Carrier | G | 84 | 6 | 3 | 9 | 1 | 8 | 3 | 2 | 84 | 107 |
| B49 | stg4831 | Mumbai | Throat | Pharyngitis | G | 84 | 6 | 3 | 9 | 1 | 8 | 3 | 2 | 84 | 107 |
| B5 | stg4831 | Mumbai | Throat | Carrier | G | 84 | 6 | 3 | 9 | 1 | 8 | 3 | 2 | 84 | 107 |
| B50 | stg4831 | Mumbai | Throat | Pharyngitis | G | 84 | 6 | 3 | 9 | 1 | 8 | 3 | 2 | 84 | 107 |
| B55 | stg4831 | Mumbai | Throat | Pharyngitis | G | 84 | 6 | 3 | 9 | 1 | 8 | 3 | 2 | 84 | 107 |
| B6 | stg4831 | Mumbai | Throat | Pharyngitis | G | 84 | 6 | 3 | 9 | 1 | 8 | 3 | 2 | 84 | 107 |
| B60 | stg4831 | Mumbai | Throat | Pharyngitis | G | 84 | 6 | 3 | 9 | 1 | 8 | 3 | 2 | 84 | 107 |
| B7 | stg4831 | Mumbai | Throat | Carrier | G | 84 | 6 | 3 | 9 | 1 | 8 | 3 | 2 | 84 | 107 |
| B8 | stg4831 | Mumbai | Throat | Carrier | G | 84 | 6 | 3 | 9 | 1 | 8 | 3 | 2 | 84 | 107 |
| B9 | stg4831 | Mumbai | Throat | Pharyngitis | G | 84 | 6 | 3 | 9 | 1 | 8 | 3 | 2 | 84 | 107 |
| B17 | stg4831 | Mumbai | Throat | Carrier | G | 85 | 6 | 3 | 9 | 11 | 22 | 3 | 2 | 85 | 107 |
| B220 | stg4831 | Mumbai | Throat | Carrier | G | 87 | 3 | 3 | 9 | 1 | 8 | 3 | 2 | 84 | 107 |
| B199 | stG4831 | Mumbai | Throat | Carrier | G | 102 | 6 | 3 | 5 | 1 | 9 | 30 | 2 | 102 | 89 |
| C100 | stg485 | Chennai | Throat | carrier | G | 100 | 4 | 2 | 4 | 2 | 9 | 22 | 16 | 100 | s |
| C111 | stg4974 | Chennai | Throat | carrier | G | 119 | 4 | 3 | 4 | 1 | 21 | 3 | 2 | 119 | s |
| C133 | stg4974 | Chennai | Throat | carrier | G | 119 | 4 | 3 | 4 | 1 | 21 | 3 | 2 | 119 | s |
| C141 | stg4974 | Chennai | Throat | carrier | G | 119 | 4 | 3 | 4 | 1 | 21 | 3 | 2 | 119 | s |
| 497 | stg6 | Mumbai | Skin | Pyoderma | G | 44 | 2 | 2 | 4 | 2 | 3 | 7 | 1 | 44 | 44 |
| B102 | stg6 | Mumbai | Throat | Carrier | G | 44 | 2 | 2 | 4 | 2 | 3 | 7 | 1 | 44 | 44 |
| B84 | stg6 | Mumbai | Throat | Pharyngitis | G | 44 | 2 | 2 | 4 | 2 | 3 | 7 | 1 | 44 | 44 |
| B93 | stg6 | Mumbai | Throat | Pharyngitis | C | 44 | 2 | 2 | 4 | 2 | 3 | 7 | 1 | 44 | 44 |
| B110 | stg6 | Mumbai | Throat | Carrier | C | 81 | 3 | 2 | 4 | 1 | 5 | 7 | 2 | 81 | 107 |
| 496 | stg6 | Mumbai | Skin | Pyoderma | G | 117 | 15 | 2 | 4 | 2 | 3 | 7 | 1 | 44 | 44 |
| 498 | stg6 | Mumbai | Skin | Pyoderma | G | 122 | 16 | 2 | 4 | 1 | 3 | 7 | 15 | 122 | 44 |
| 499 | stg6 | Mumbai | Skin | Pyoderma | G | 123 | 16 | 2 | 4 | 2 | 3 | 7 | 1 | 44 | 44 |
| 500 | stg6 | Mumbai | Skin | Pyoderma | G | 123 | 16 | 2 | 4 | 2 | 3 | 7 | 1 | 44 | 44 |
| 501 | stg6 | Mumbai | Throat | Pharyngitis | G | 124 | 3 | 2 | 4 | 1 | 4 | 1 | 2 | 124 | 107 |
| 502 | stg6 | Mumbai | Throat | Carrier | G | 124 | 3 | 2 | 4 | 1 | 4 | 1 | 2 | 124 | 107 |
| C66 | stg643 | Chennai | Throat | Tonsillitis | G | 112 | 2 | 2 | 4 | 2 | 3 | 7 | 7 | 44 | 44 |
| C67 | stg643 | Chennai | Throat | Tonsillitis | G | 112 | 2 | 2 | 4 | 2 | 3 | 7 | 7 | 44 | 44 |
| C123 | stg643 | Chennai | Throat | carrier | G | 120 | 2 | 2 | 12 | 2 | 3 | 7 | 7 | 44 | 44 |
| B124 | stg652 | Mumbai | Throat | Pharyngitis | G | 70 | 3 | 10 | 4 | 1 | 5 | 10 | 2 | 70 | 107 |
| B69 | stg652 | Mumbai | Throat | Pharyngitis | G | 70 | 3 | 10 | 4 | 1 | 5 | 10 | 2 | 70 | 107 |
| C119 | stg652 | Chennai | Throat | carrier | G | 70 | 3 | 10 | 4 | 1 | 5 | 10 | 2 | 70 | 107 |
| C88 | stg652 | Chennai | Throat | Pharyngitis | n.d. | 114 | 3 | 10 | 4 | 2 | 5 | 10 | 2 | 70 | 107 |
| C95 | stg652 | Chennai | Skin | wound swab | n.d. | 115 | 3 | 2 | 1 | 2 | 5 | 10 | 2 | 70 | 107 |
| B10 | stg653 | Mumbai | Throat | Pharyngitis | G | 95 | 2 | 2 | 5 | 2 | 3 | 7 | 13 | 44 | 44 |
| B127 | stg653 | Mumbai | Throat | Carrier | G | 95 | 2 | 2 | 5 | 2 | 3 | 7 | 13 | 44 | 44 |
| B151 | stg653 | Mumbai | Throat | Carrier | C | 95 | 2 | 2 | 5 | 2 | 3 | 7 | 13 | 44 | 44 |
| C116 | stg653 | Chennai | Throat | carrier | G | 95 | 2 | 2 | 5 | 2 | 3 | 7 | 13 | 44 | 44 |
| C69 | stg653 | Chennai | Throat | Pharyngitis | n.d. | 95 | 2 | 2 | 5 | 2 | 3 | 7 | 13 | 44 | 44 |
| B202 | stG653 | Mumbai | Throat | Carrier | G | 104 | 5 | 3 | 5 | 1 | 6 | 2 | 1 | 104 | s |
| B126 | stg6792 | Mumbai | Throat | Pharyngitis | G | 66 | 3 | 3 | 4 | 2 | 9 | 22 | 2 | 66 | 66 |
| B150 | stg6792 | Mumbai | Throat | Carrier | C | 66 | 3 | 3 | 4 | 2 | 9 | 22 | 2 | 66 | 66 |
| B66 | stg6792 | Mumbai | Throat | Carrier | G | 66 | 3 | 3 | 4 | 2 | 9 | 22 | 2 | 66 | 66 |
| C129 | stg6792 | Chennai | Throat | carrier | G | 66 | 3 | 3 | 4 | 2 | 9 | 22 | 2 | 66 | 66 |
| C63 | stg6792 | Chennai | Throat | Tonsillitis | n.d. | 66 | 3 | 3 | 4 | 2 | 9 | 22 | 2 | 66 | 66 |
| C98 | stg6792 | Chennai | Cellular fluid | cellular fluid | n.d. | 66 | 3 | 3 | 4 | 2 | 9 | 22 | 2 | 66 | 66 |
| B67 | stg6792 | Mumbai | Throat | Carrier | G | 95 | 2 | 2 | 5 | 2 | 3 | 7 | 13 | 44 | 44 |
| C65 | stg6792 | Chennai | Throat | Tonsillitis | G | 111 | 3 | 3 | 4 | 2 | 9 | 25 | 2 | 66 | 66 |
| C82 | stg6792 | Chennai | Throat | Pharyngitis | n.d. | 111 | 3 | 3 | 4 | 2 | 9 | 25 | 2 | 66 | 66 |
| C86 | stg6792 | Chennai | Throat | Tonsillitis | G | 111 | 3 | 3 | 4 | 2 | 9 | 25 | 2 | 66 | 66 |
| C90 | stg6792 | Chennai | Throat | Pharyngitis | n.d. | 111 | 3 | 3 | 4 | 2 | 9 | 25 | 2 | 66 | 66 |
| C91 | stg6792 | Chennai | Throat | Pharyngitis | n.d. | 111 | 3 | 3 | 4 | 2 | 9 | 25 | 2 | 66 | 66 |
| C121 | stg840 | Chennai | Throat | carrier | G | 34 | 3 | 7 | 4 | 1 | 14 | 15 | 10 | 34 | 34 |
| B255 | stg866 | Mumbai | Throat | Carrier | G | 15 | 3 | 3 | 2 | 2 | 9 | 8 | 2 | 15 | 66 |
| B31 | stg866 | Mumbai | Throat | Pharyngitis | G | 89 | 13 | 3 | 5 | 1 | 9 | 26 | 2 | 89 | 89 |
| B32 | stg866 | Mumbai | Throat | Pharyngitis | G | 89 | 13 | 3 | 5 | 1 | 9 | 26 | 2 | 89 | 89 |
| B45 | stg866 | Mumbai | Throat | Pharyngitis | G | 89 | 13 | 3 | 5 | 1 | 9 | 26 | 2 | 89 | 89 |
| B46 | stg866 | Mumbai | Throat | Pharyngitis | G | 89 | 13 | 3 | 5 | 1 | 9 | 26 | 2 | 89 | 89 |
| B47 | stg866 | Mumbai | Throat | Pharyngitis | G | 89 | 13 | 3 | 5 | 1 | 9 | 26 | 2 | 89 | 89 |
| B85 | stg866 | Mumbai | Throat | Carrier | G | 89 | 13 | 3 | 5 | 1 | 9 | 26 | 2 | 89 | 89 |
| C79 | stg866 | Chennai | Throat | Tonsillitis | G | 89 | 13 | 3 | 5 | 1 | 9 | 26 | 2 | 89 | 89 |
| C81 | stg866 | Chennai | Throat | Tonsillitis | G | 89 | 13 | 3 | 5 | 1 | 9 | 26 | 2 | 89 | 89 |
| C85 | stg866 | Chennai | Throat | Tonsillitis | G | 89 | 13 | 3 | 5 | 1 | 9 | 26 | 2 | 89 | 89 |
| B210 | stg866 | Mumbai | Throat | Carrier | G | 92 | 13 | 3 | 14 | 1 | 22 | 26 | 14 | 92 | s |
| B212 | stg866 | Mumbai | Throat | Carrier | G | 93 | 13 | 3 | 5 | 1 | 8 | 26 | 2 | 89 | 89 |
| B111 | stg866 | Mumbai | Throat | Carrier | G | 126 | 13 | 3 | 15 | 1 | 9 | 26 | 2 | 89 | 89 |
| B184 | stgL265 | Mumbai | Throat | Carrier | G | 15 | 3 | 3 | 2 | 2 | 9 | 8 | 2 | 15 | 66 |
| B215 | stGL265 | Mumbai | Throat | Pharyngitis | G | 44 | 2 | 2 | 4 | 2 | 3 | 7 | 1 | 44 | 44 |
| B217 | stgL265 | Mumbai | Throat | Carrier | G | 101 | 2 | 2 | 4 | 15 | 3 | 7 | 1 | 44 | 44 |
| B201 | stgLP2 | Mumbai | Throat | Carrier | G | 103 | 2 | 2 | 4 | 2 | 3 | 7 | 2 | 44 | 44 |
| B152 | stgm22 | Mumbai | Throat | Carrier | C | 34 | 3 | 7 | 4 | 1 | 14 | 15 | 10 | 34 | 34 |
| B65 | stgm22 | Mumbai | Throat | Carrier | G | 34 | 3 | 7 | 4 | 1 | 14 | 15 | 10 | 34 | 34 |
| B128 | stgm22 | Mumbai | Throat | Carrier | G | 94 | 3 | 7 | 13 | 1 | 14 | 15 | 10 | 34 | 34 |
| B129 | stgm22 | Mumbai | Throat | Carrier | G | 125 | 3 | 10 | 4 | 1 | 14 | 15 | 10 | 34 | 34 |
