# Supplementary material for: Recombination Drives Genetic Diversification of Streptococcus dysgalactiae Subspecies equisimilis in a Region of Streptococcal Endemicity
Source: PLoS One. 2011 Aug 3;6(8):e21346. doi: 10.1371/journal.pone.0021346 (PMC3153926; doi:10.1371/journal.pone.0021346)
Supplement: Table S2 — Novel MLST alleles in the Indian SDSE population. (DOC) [file pone.0021346.s006.doc]

**Table S2**. Novel MLST alleles in the Indian SDSE population.

| **Allele** | **SDSE allele** | ***S. pyogenes* allele** |
| --- | --- | --- |
| *gki* | 13 | 3 |
|  | 15 | 67 |
|  | 16 | 38 |
| *gtr* | 11 | 31 |
|  | 12 | 3 |
| *murI* | 12 | 58 |
|  | 13 | Novel |
|  | 14 | Novel |
|  | 15 | 4 |
| *mutS* | 11 | Novel |
|  | 12 | Novel |
|  | 13 | Novel |
|  | 14 | 18 |
|  | 15 | Novel |
|  | 16 | Novel |
| *recP* | 21 | 21 |
|  | 22 | *S. agalactiae* NEM316 |
|  | 24 | Novel |
|  | 25 | Novel |
| *xpt* | 23 | Novel |
|  | 24 | Novel |
|  | 25 | Novel |
|  | 26 | Novel |
|  | 27 | Novel |
|  | 28 | Novel |
|  | 29 | Novel |
|  | 30 | Novel |
| *atoB* | 13 | Novel |
|  | 14 | Novel^a^ |
|  | 15 | Yqi_14 |
|  | 16 | Novel |
|  | 17 | Novel |

^a^*ato14* is 100% identical to an allele from *S. pyogenes* NZ131 and other *S. pyogenes* genomes that are not part of the current *S. pyogenes* MLST scheme. However phylogenetic analysis demonstrates that *ato13* is more closely related to other SDSE *ato* alleles than other *S. pyogenes* alleles. i.e. NZ31 and other *S. pyogenes* strains have acquired the *ato14* SDSE allele.
