# Supplementary material for: Recombination Drives Genetic Diversification of Streptococcus dysgalactiae Subspecies equisimilis in a Region of Streptococcal Endemicity
Source: PLoS One. 2011 Aug 3;6(8):e21346. doi: 10.1371/journal.pone.0021346 (PMC3153926; doi:10.1371/journal.pone.0021346)
Supplement: Table S3 — Recombination and mutation in SDSE. (DOC) [file pone.0021346.s007.doc]

**Table S3**. Recombination and mutation in SDSE.

|  |  | **SLV ST pair** | |  |  |  |  |
| --- | --- | --- | --- | --- | --- | --- | --- |
| **Clonal Complex** | **locus** | **ST** | **ST** | **Allele 1** | **Allele 2** | **nt variant sites** | **R orM ^a^** |
| 34 | *gtr* | 34 | 125 | 7 | 10 | 3 | R |
|  | *murI* | 94 | 34 | 4 | 13 | 6 | R |
| 44 | *atoB* | 44 | 103 | 1 | 2 | 1 | M |
|  | *atoB* | 44 | 116 | 1 | 14 | 5 | R |
|  | *atoB* | 44 | 112 | 1 | 7 | 3 | R |
|  | *atoB* | 44 | 86 | 1 | 17 | 34 | R |
|  | *atoB* | 98 | 95 | 1 | 13 | 4 | R |
|  | *gki* | 44 | 123 | 2 | 16 | 52^b^ | R |
|  | *gki* | 44 | 117 | 2 | 15 | 50^b^ | R |
|  | *murI* | 44 | 98 | 4 | 5 | 8 | R |
|  | *murI* | 103 | 99 | 4 | 5 | 8 | R |
|  | *mutS* | 44 | 101 | 2 | 15 | 1 | R |
|  | *mutS* | 44 | 96 | 2 | 12 | 1 | R |
| 66 | *gki* | 66 | 121 | 3 | 2 | 2 | R |
|  | *xpt* | 66 | 111 | 22 | 25 | 1 | M |
| 70 | *gtr* | 82 | 115 | 10 | 2 | 3 | R |
|  | *murI* | 82 | 115 | 4 | 1 | 1 | R |
|  | *mutS* | 114 | 70 | 2 | 1 | 1 | R |
| 84 | *gki* | 84 | 87 | 6 | 3 | 2 | R |
|  | *murI* | 107 | 106 | 4 | 9 | 9 | R |
|  | *xpt* | 106 | 87 | 7 | 3 | 19 | R |
| 89 | *murI* | 89 | 126 | 5 | 15 | 107^b^ | R |
|  | *recP* | 93 | 89 | 8 | 9 | 1 | R |
| 97 | *recP* | 97 | 108 | 8 | 25 | 8 | R |

^a^Recombination (R) or mutation (M)

^b^Descendent allele predicted to have been acquired from *S. pyogenes*.
