# Supplementary material for: Recombination Drives Genetic Diversification of Streptococcus dysgalactiae Subspecies equisimilis in a Region of Streptococcal Endemicity
Source: PLoS One. 2011 Aug 3;6(8):e21346. doi: 10.1371/journal.pone.0021346 (PMC3153926; doi:10.1371/journal.pone.0021346)
Supplement: Table S4 — Emm -types associated with multiple Sequence Types. (DOC) [file pone.0021346.s008.doc]

**Table S4.** *Emm*-types associated with multiple Sequence Types.

| ***Emm*** | **ST** | **CC_slv_** | **CC_dlv_** |
| --- | --- | --- | --- |
| *stc1400* | 66,88, 90 | 66,88, 90 | 66, 107 |
| *stc36* | 15, 44, 86, 96,97, 98, 109,116 | 15, 44, 97, 109 | 44, 66, 97, s |
| *stc74a* | 29, 83 | 29, 83 | 29, 107 |
| *stg245* | 15, 29, 44, 81, 82, 83, 110, | 15, 29, 44, 81, | 29, 44, 66, 107 |
| *Stg4222* | 105, 106 | 84, 105 | 107 |
| *stg480* | 34, 44, 98, 99,121 | 34, 44, 66 | 34, 44, 66 |
| *stg4831* | 84, 85, 87, 102 | 84, 102 | 102, 89 |
| *stg6* | 44, 81, 117, 122, 123, 124, | 44, 81, 122, 124 | 44, 107 |
| *stg643* | 112, 120 | 44 | 44 |
| *stg652* | 70, 114, 115 | 70 | 107 |
| *stg653* | 95, 104 | 44, 104 | 44, s |
| *stg6972* | 66, 95, 111 | 44, 66 | 44, 66 |
| *stg866* | 15, 89, 92, 93, 126 | 15, 89, 92 | 66, 89, s |
| *stgL265* | 15, 44, 101 | 15, 44 | 66, 44 |
| *stgm22* | 34, 94, 125 | 34 | 34 |
